# Accurate influenza forecasts using type-specific incidence data for small geographic units

**DOI:** 10.1371/journal.pcbi.1009230

**Published:** 2021-07-29

**Authors:** James Turtle, Pete Riley, Michal Ben-Nun, Steven Riley

**Affiliations:** 1 Infectious Disease Group, Predictive Science Inc., San Diego, California, United States; 2 MRC Centre for Outbreak Analysis and Modelling, Department of Infectious Disease Epidemiology, School of Public Health, Imperial College London, United Kingdom; University of Notre Dame, UNITED STATES

## Abstract

Influenza incidence forecasting is used to facilitate better health system planning and could potentially be used to allow at-risk individuals to modify their behavior during a severe seasonal influenza epidemic or a novel respiratory pandemic. For example, the US Centers for Disease Control and Prevention (CDC) runs an annual competition to forecast influenza-like illness (ILI) at the regional and national levels in the US, based on a standard discretized incidence scale. Here, we use a suite of forecasting models to analyze type-specific incidence at the smaller spatial scale of clusters of nearby counties. We used data from point-of-care (POC) diagnostic machines over three seasons, in 10 clusters, capturing: 57 counties; 1,061,891 total specimens; and 173,909 specimens positive for Influenza A. Total specimens were closely correlated with comparable CDC ILI data. Mechanistic models were substantially more accurate when forecasting influenza A positive POC data than total specimen POC data, especially at longer lead times. Also, models that fit subpopulations of the cluster (individual counties) separately were better able to forecast clusters than were models that directly fit to aggregated cluster data. Public health authorities may wish to consider developing forecasting pipelines for type-specific POC data in addition to ILI data. Simple mechanistic models will likely improve forecast accuracy when applied at small spatial scales to pathogen-specific data before being scaled to larger geographical units and broader syndromic data. Highly local forecasts may enable new public health messaging to encourage at-risk individuals to temporarily reduce their social mixing during seasonal peaks and guide public health intervention policy during potentially severe novel influenza pandemics.

## Author summary

Forecasting influenza is a public health priority because it allows better planning by both policy makers and healthcare facilities. Most seasonal influenza forecasting is currently undertaken for large geographical areas and is based on the number of people who have the signs and symptoms of influenza. Different types of models can provide roughly equivalent forecasts for these data: models that use the opinion of experts, statistical models, and models that reflect the underlying biology. Newly developed data streams are resolving ever smaller spatial scales, but the impacts of coupling and smaller resolution on type-specific forecasting have not been well established.

In addition to the use of local point-of-care type-specific influenza data, we assess the effects of county-to-county coupling and specific humidity on forecast outcomes. Here, we show that if the data are available for smaller geographical areas and are from actual tests for influenza, they can be forecasted accurately by models that represent the underlying biological mechanisms. Further, we add to growing evidence that mechanistic models perform better on type-specific data than they do on symptom-based data, which can be triggered by a number of respiratory viruses and/or influenza types.

## Introduction

Influenza infections cause substantial morbidity and mortality across all geographical areas and sociodemographic groups [1]. Forecasting pipelines—of data and associated analytics—can create knowledge of current and likely future incidence and therefore have high potential value to individuals and health planners [2], potentially leading to at-risk individuals choosing to modify their behaviour to reduce their individual risk when incidence is high [3]. Some health officials are already working to incorporate forecasts into decision making [4], and health systems could more efficiently manage scarce resources such as intensive care services that are impacted by local influenza incidence but also utilized by many different healthcare pathways [5]. More generally, seasonal influenza is an ideal modelling case study for respiratory virus pandemics. Recent pandemics (influenza—2009, SARS-CoV-2—2020) have demonstrated the difficult health and socioeconomic decisions that must be made during a novel virus outbreak, making forecasting a key priority to support rapid investment decisions and optimization of available resources [6, 7].

Current forecasting pipelines for influenza focus on the use of syndromic data for large geographically diffuse populations [8]. For example, the US Centers for Disease Control and Prevention (CDC) forecasting challenge produces public forecasts of influenza-like illness rates at regional and national scales for the US, based on a long-running national syndromic surveillance system that has proven robust during a pandemic [9]. Influenza forecasters use a variety of methods that can be categorized by their underlying model: mechanistic, statistical, and crowd-sourced. Mechanistic models use epidemiological first principles to approximate disease transmission dynamics. In practice this often takes the form of compartmental differential equations describing a metapopulation [10–14]. Statistical models [15–19] use patterns and tendencies in the data-stream history to predict future data values. This includes many forms of implementation including: various types of regression, machine-learning, and time-series methods. Crowd-sourced models present participants with current data and ask them to manually construct a forecast. In practice, the ‘crowd’ generally consists of volunteer experts who update their forecasts weekly [20, 21]. Of course, complete model descriptions are much more nuanced with many mechanistic models being housed in a probabilistic framework [10–14], crowd-sourced model selection by machine learning [22], combination mechanistic and statistic models [23], ensembles of automated models [24, 25], multi-team ensembles [2], and models that incorporate social media metrics [19, 26] to name a few.

In the United States, syndromic influenza-like illness (ILI) data is available at the national, regional, and state levels [27] in a consistent and computer-readable format. At smaller spatial scale, influenza data is often publicly available for large cities and/or counties, but in inconsistent data-types (outpatient ILI, emergency-room ILI, laboratory positive tests, etc) and often not in a computer-quantifiable format (i.e. PDF). One group has collected influenza data from the public health departments of many counties and cities in the U.S. [28], but presumably at significant cost. Possibly due to this lack of availability and coverage, there have been relatively few studies that attempt to forecast at the county [12, 28, 29] or city [26, 30–32] level. Of those, only [28] applies a model broadly without limiting focus to one or two areas per country.

Unlike existing data sources, the dataset used here provides access to spatial resolution as precise as zip code. The Quidel point of care dataset has been used for one other study [33], which focused on U.S. regional level nowcasting and forecasting. Point-of-care diagnostic results are becoming increasingly available to researchers. BioFire Diagnostics recently announced a similar dataset [34] that includes influenza sub-type results in addition to type data.

Here, we consider the possibility that alternate data streams may be much more closely related to the underlying biology and hence be inherently more forecastable using mechanistic models. Specifically, that more-local and pathogen-specific data may permit more accurate forecasting pipelines with mechanistic models than are currently possible using more geographically aggregated syndromic data [8, 35] or low resolution type-specific data cross referenced with local syndromic proxy data [28]. We apply a set of previously validated models [11] to incidence data from geolocated point-of-care diagnostic devices [36], and use a skill scoring system that is analogous to that used thus far [8, 37] for the CDC prospective competition to compare the forecastability of alternate data streams.

## Materials and methods

### Deployment of test machines and capture of test data

Our data are gathered from a near-real time network of benchtop Sofia and Sofia 2 POC diagnostic machines (Quidel, San Diego, USA). At the start of the study period in July 2016, the network consisted of 1,676 machines located in physician offices (880), hospitals (257), urgent care clinics (427) and other/not reported (112) settings. At the end of the study period (July 2019) the network had grown to 12,345 machines with 5,736 in physician offices, 1,206 in hospitals, 1,194 in urgent care clinics, and 4,209 in other/not reported settings. The standard kit tests for influenza A and B. Data are automatically transmitted to Quidel. Reporting cadence can vary depending on device connectivity, but most devices report results within a day of testing.

### Selection of counties and clusters

**Fig 1:**
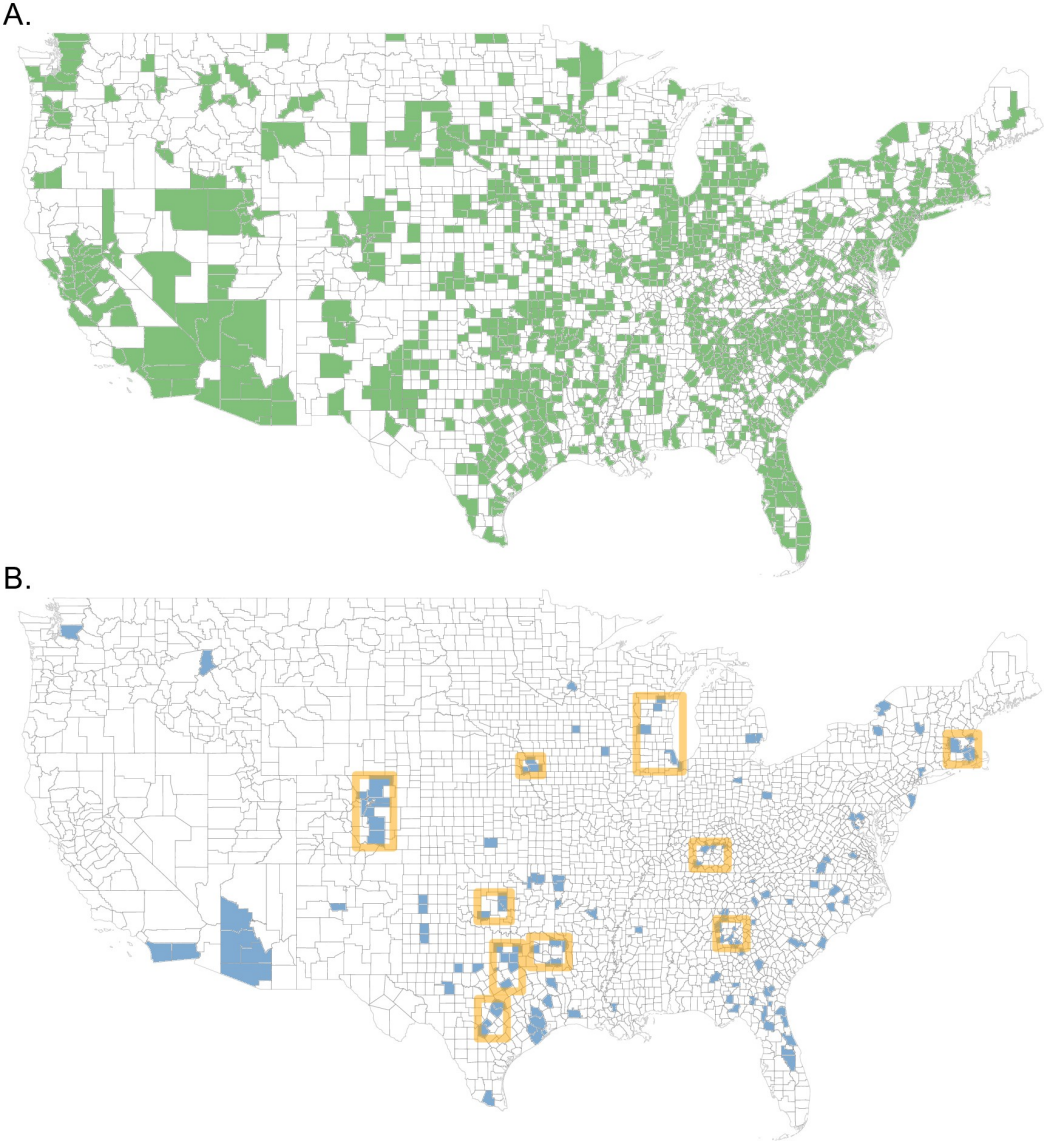
County filtering and clustering. (A) All counties with minimum one test result from week 27, 2016 to week 26, 2019. (B) Counties with at least 250 specimens tested for the seasons 2016–17, 2017–18, and 2018–19 and 40 weeks of non-zero reporting for the seasons 2016–17 and 2017–18 (as calculated on week 3, 2019). Square orange borders outline the 10 county clusters considered in this study. The map base layer was sourced from the U.S. Census Bureau [39]

Individual strain tests were sorted into weekly totals for each county represented in the dataset. Of the approximately 3,240 counties and county equivalents in the United States, 1,258 are represented in this dataset (see Fig 1A). However, to omit sparse data streams, counties that did not report for at least 40 weeks in both the 2016–17 and 2017–18 seasons were discarded. Next, counties with less than 250 specimens tested in any single season of 2016–17, 2017–18, or 2018–19 were discarded. These requirements reduced the number of considered counties from 1,258 to 136. This county filtering process was based on data reported up to and including week 3 of 2019. Throughout this manuscript, weeks are numbered according to the Morbidity and Mortality Weekly Report (MMWR) definition [38].

To identify localized groups within the 136 qualifying counties, we applied a k-means clustering algorithm for the number of clusters *k*. Cluster distances were based on county population centroids *c* (lat/lon). For a single cluster *a*_*j*_ with *N* counties and geographic cluster centroid *b*_*j*_, we define the intra-cluster distance as the sum of squares

$$a_{j}=\sum_{i=1}^{N}{∥c_{i}-b_{j}∥}^{2}$$

where *c*_*i*_ is the centroid of the *i*-th county of cluster *j*. The k-means algorithm was applied while varying *k* over the interval [2, 60]. The total intra-cluster sum of squares (over all clusters) stopped decreasing at approximately *k* = 38. So we chose *k* = 38. Smaller, sparsely spaced clusters were removed by requiring *N* > = 4 and *a*_*j*_/*N* < = 0.5.

The one exception to these rules is the Atlanta cluster. Most Atlanta counties do not pass the incidence minimums for the 2016–17 season. However, county coverage for 2017–18 and 2018–19 is the best of any large metropolitan area in the United States, so Atlanta was included for these seasons.

This process resulted in 10 county clusters, covering 57 counties as described in S2 Table. The 136 filtered counties and resulting county clusters are illustrated in Fig 1B.

The cutoff values for county inclusion were chosen to insure a sampling of county clusters with which to test the intra-county coupling across all three seasons. These decisions reduced the total number of counties (1258) drastically to only 136. However, it should be noted that the number of counties meeting these cutoffs (250 specimens and 40 reporting weeks per season) has increased significantly each season (see Table 1) from 136 to 541. A map of eligible counties by season appears in S1 Fig. Furthermore, the population encompassed by filtered counties increased from 76 million in 2016–17 to 181 million in 2018–19, providing representative data for over half of the country’s population.

**Table 1:** Filtered county totals by season.

Season	No. counties	Population (millions) encompassed
2016–17	136	76
2017–18	281	109
2018–19	541	181

Total number of counties with at least 250 specimens tested and 40 weeks reported in a season. The right column is the total population of the filtered counties. These totals were calculated following the end of the 2018–19 season.

For each county of the 10 clusters, data was compiled to weekly totals for both Total Specimens (TS) and specimens that were Positive for A (PA). Weekly cluster data resulted from a sum of the constituent counties.

### Mechanistic modelling framework

The following sections offer a summary of the model framework, covariate data collection, and fitting procedure used for this work. These concepts were executed using the software package Dynamics of Interacting Community Epidemics (DICE). DICE is a publicly available R-package available for download with documentation and examples [40].

The foundation of our model is the common Susceptible-Infectious-Recovered (SIR) set of equations [41]

$$\begin{array}{cc} \frac{dS_{i}}{dt}= & \;-\lambda_{i}\left(t\right)S_{i},\\ \frac{dI_{i}}{dt}= & \;\lambda_{i}\left(t\right)S_{i}-\frac{I_{i}}{T_{g}},\\ \frac{dR_{i}}{dt}= & \;\frac{I_{i}}{T_{g}}. \end{array}$$

Here the state variables are number of susceptibles *S*, infectious *I*, and recovered *R* in the *i*th physical region. The equations are completed by generation time *T*_*g*_ and force of infection λ. The model is applied to a single season with the assumption that the entire population is susceptible at start of season. Included in λ are terms to modulate the force of infection for specific humidity and county coupling

$$\lambda_{i}\left(t\right)=\sum_{j=1}^{D}\beta_{j}\left(t\right)m_{ij}\frac{\sum_{l=1}^{D}m_{lj}I_{l}}{\sum_{p=1}^{D}m_{pj}N_{p}}.$$

The transmitting-contact rate *β*_*j*_(*t*) pertains to transmission dynamics internal to county *j*, where *D* is the total number of coupled counties. The mobility matrix *m*_*ij*_ describes the probability that a contact made by an individual in *i* was with an individual from *j* [42]. The probability of inter-county contacts is proportionate to the mobility kernel

$$\kappa \left(r_{ij}\right)=\frac{1}{1+{\left(r_{ij}/s_{d}\right)}^{\gamma }}$$

that is a variation of the offset power function. Here, the distance *r*_*ij*_ is calculated as the great-circle distance between counties’ population centroids. Saturation distance *s*_*d*_ and the exponent *γ* are fit parameters. The mobility matrix is population weighted and row-normalized

$$m_{ij}=N_{j}\kappa \left(r_{ij}\right)\frac{1}{\sum_{k}N_{k}\kappa \left(r_{ik}\right)}.$$

Following the functional form in [43], the transmitting-contact rate in region *i* becomes time-dependent when modulated by specific humidity *q*_*i*_(*t*)

$$\beta_{i}\left(t\right)=\beta_{0i}(1+\Delta_{Ri}\cdot e^{-a_{i}\cdot q_{i}\left(t\right)}).$$

The basic reproduction number *R*_0_ = *T*_*g*_ *β*_0*i*_, specific humidity scaling Δ_*Ri*_, and specific humidity power *a*_*i*_ are fit parameters. When Δ_*Ri*_ → 0, the transmitting-contact rate reverts to a constant value *β*_0*i*_.

All results presented here pertain to forecasting cluster weekly incidence, where cluster incidence is the sum of the counties incidence. The spatial aspect of the model is characterized in three ways: direct, uncoupled, and coupled. The self-describing ‘direct’ fit matches the model directly to cluster totals. When applying the uncoupled model to a cluster, each constituent county is fit independently. The individual county forecasts are then aggregated to produce a cluster forecast. The coupled approach simultaneously fits the coupled model to all constituent counties.

Specific humidity data was taken from the National Centers for Environmental Prediction (NCEP)/National Center for Atmospheric Research (NCAR) Reanalysis-2 project [44]. This provides climate model evaluations 1979-present on a 1.875° spatial grid. The reanalysis-2 spatial grid is layered with GADM (the Database of Global Administrative Areas) county definitions [45] and the SocioEconomic Data and Applications Center (SEDAC) population densities [46] to produce weekly population-weighted averages for specific humidity in each county.

### Model fitting

Here we define incidence as the population count that was ILI symptomatic (TS) or influenza infectious (PA) and presented to a reporting clinic for testing. Model equations are applied to the full population, however model incidence-reported IRm is returned from the model as a proportion of weekly new infectious as

$$\text{IRm}=B+p_{C}\int_{t_{i-1}}^{t_{i}}\lambda \left(t\right)S\left(t\right)dt.$$

The proportion clinical *p*_*C*_ scaling accounts for both the portion of the population covered by reporting clinics and the proportion of infectious that sought medical evaluation/treatment. In this framework *t*_*i*_ is defined as the Saturday that ends week *i*, and *t*_*i*−1_ is the previous Saturday. A baseline incidence *B* is introduced as a phenomenological parameter that accounts for year-round testing and, particularly, early-season weeks where the incidence trend is not yet exponential. *B* and *p*_*C*_ are both fit parameters.

In the fitting procedure, the objective function is a Poisson based, Log-Likelihood (LLK). For the *i*-th week of data, we calculate the probability that the data incidence reported IRd_*i*_ is a Poisson expression of model incidence reported IRm_*i*_

$$P(x={\text{IRd}}_{i})=\text{Pois}({\text{IRm}}_{i}).$$

The model likelihood is then a product over *N* weeks of *P*(*x* = IRd_*i*_), and results in an LLK of

$$\text{LLK}=\sum_{i}^{N}w_{i}({\text{IRd}}_{i}\cdot \log\left({\text{IRm}}_{i}\right)-{\text{IRm}}_{i}-\log\left({\text{IRd}}_{i}\right)).$$

Here, *w*_*i*_ is used to give zero weight to weeks where the data has been designated ‘no data’. The following is a summary description of how the spatial aspect of the model effects the fitting procedure. For the direct model, a single LLK fits the model to cluster totals. For the uncoupled model, we independently fit the model to each county and then aggregate model results to cluster forecasts. For the coupled model, cluster LLK is calculated as a population-weighted average of the individual county LLKs, and thus the counties are fit simultaneously in a single fit procedure.

The fit procedure is a Metropolis-Hastings type Markov Chain Monte Carlo (MCMC) process [47] with adaptive step size to map the likelihood minima in parameter space. This procedure was implemented in Fortran and is accessed through the R wrapper, all of which is included in the linked GitHub repository. For each MCMC fit, we processed three randomly-initialized chains, each for 2 × 10^6^ steps, and returned 10^4^ parameter sets (evenly distributed from the second half of the chain). Of the three chains, we chose the one with the lowest LLK minimum. By using 3 chains starting in different parts of parameter space, we avoid the rare event where a chain gets ‘stuck’ in a local minima of the objective function. Due to the lack of sufficient historic data and resulting model-fit posteriors, we do not attempt to use informed parameter priors. Therefore, all parameter priors are uniform.

For each of the three seasons (2016–17, 2017–18, 2018–19) we fit the data from week 27 to *x*, where *x* covered the weeks [36, …, 52, 1, …, 18], and projected the model forward 1 to 10 weeks. This resulted in 1- to 10-week forecasts for each of the evaluated weeks [46, …, 52, 1, …, 19]. At each week we applied 6 model variants stemming from combinations of spatial organization (direct, uncoupled, coupled) and *β*-modulation (fixed *β*, humidity-modulated *β*).

### Forecast scoring

For each forecast target, our model produces a distribution of predicted outcomes. This distribution is binned for evaluation based on the scoring system used by the CDC Influenza Challenge for the 2016–17, 2017–18 and 2018–19 seasons [37]. The maximum range is separated into 130 equally sized bins with a 131st bin for all values over the maximum previously observed. In this scoring system, higher magnitude seasons tend to score lower. As a simple correction for this, we re-scaled the bins for TS and PA so that both metrics peak in the same bin as CDC %ILI for the encompassing region (see ‘CDC_Parent’ in S2 Table). Although each cluster was generally much smaller than its encompassing CDC state/region, we suggest that CDC %ILI is the most spatially consistent and comprehensive dataset to perform this scaling with. Re-scaled bins for the San Antonio/Austin cluster, 2016–17 season appear as an example in S2 Fig. Forecast probabilities for the observed bin (red shading) as well as the 5 pre- and pro-ceeding bins (yellow shading) are summed to produce forecast skill. Forecast score is the natural log of skill, with scores below -10 set to -10. For example, if we assigned a total probability of 20% to bins in the skill window, then the score would be ln(0.2) = −1.6. Note that due to the work of Bracher [48] and the response [49], starting with the 2019–20 influenza season, the CDC Influenza challenge defines forecast skill as only the probability in the observed bin. Here we primarily show results from the 11-bin skill window for consistency with matching Influenza Challenge seasons. However, some single bin scores are included in the supplementary material for comparison.

### Null models

For a given season, cluster, week, and data metric, Null models were evaluated using data from the same cluster, metric, and week, but different seasons. The most basic Null model ‘N.pt’ is a point forecast calculated as the mean of the Null data. The ‘N.d’ model is a distribution fit to the same data. For intensity targets we use a log normal distribution and for timing targets a normal distribution. However, both of these models suffer from the same problem: due to the small number of seasons in the dataset, the model is typically fit to only 2 data points. The third Null model ‘N.d5’ was designed to mitigate this problem. For a given forecast week, ‘N.d5’ uses the historic data from a 5-week window including the forecast week as well as the 2 pre- and pro-ceeding weeks to fit a log normal distribution. As an example, for epidemic week 4 of 2017 the data from epidemic weeks 2, 3, 4, 5, and 6 for years 2018 and 2019 are fit to a log normal. This distribution is then intensity-binned to serve as the N.d5 model forecast.

## Results

**Fig 2:**
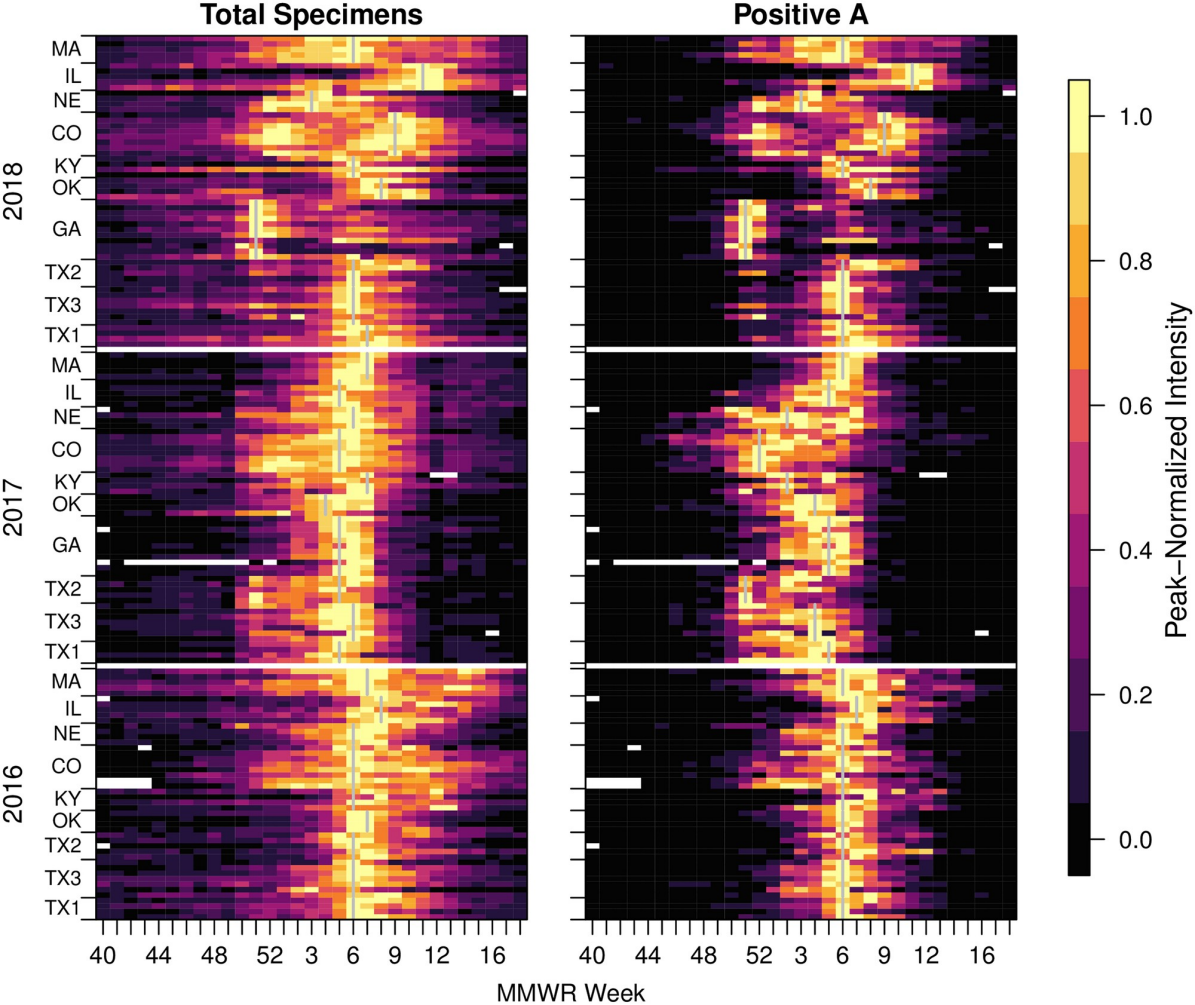
Incidence for each cluster for syndromic (total specimens, LHS) and specimens positive for A (RHS). Each row of rectangles represents a single county with counties grouped into clusters (bounded by tick marks) and then into seasons. Seasons are indicated by start year on the y-axis (with a blank row between years). Within seasons, clusters are ordered by latitude and within clusters, counties are also ordered by latitude. Cluster peak weeks are marked with a gray vertical line. In this figure, the incidence for each week is normalized for each county and year using the peak value for that year (Legend, RHS). White indicates ‘no data’.

We obtained 3,258,166 specimen results from 12,227 point-of-care (POC) machines in the United States starting from week 27, 2016 through week 26, 2019. These data were down-selected to obtain clusters of nearby counties with at least 250 tested specimens in any season, resulting in 10 clusters, capturing: 57 counties (S2 Table); 1,061,891 total specimens; and 173,909 specimens positive for Influenza A (Fig 2). Where no data was reported and there was no temporal overlap of devices, we designated that week as ‘no data’ rather than a zero. A cluster containing metropolitan Atlanta was just below the average season requirements for 2016–17 but had very high coverage for 2017–18 and 2018–19 and was thus included.

Total specimens (TS) and specimens positive for influenza A (PA) both showed broadly similar dynamics, however, there were some intriguing differences between them (Fig 2). As would be expected, the more specific PA data had a clearer difference between off-season and in-season. Also, regardless of noise during periods of low activity, the county-level PA time series produced sharper epidemics, with 6.05 weeks average duration at or above half maximum incidence compared with 8.84 weeks for the TS data. For clusters, the peaks became slightly wider with a width at half peak for PA averaging 6.48 weeks and TS averaging 9.57 weeks. The TS data showed a high correlation with influenza-like illness data for the enclosing CDC region (overall average Pearson coefficient 0.96, S1 Table). Additionally, S2 Fig serves as an example illustration of this similarity.

**Fig 3:**
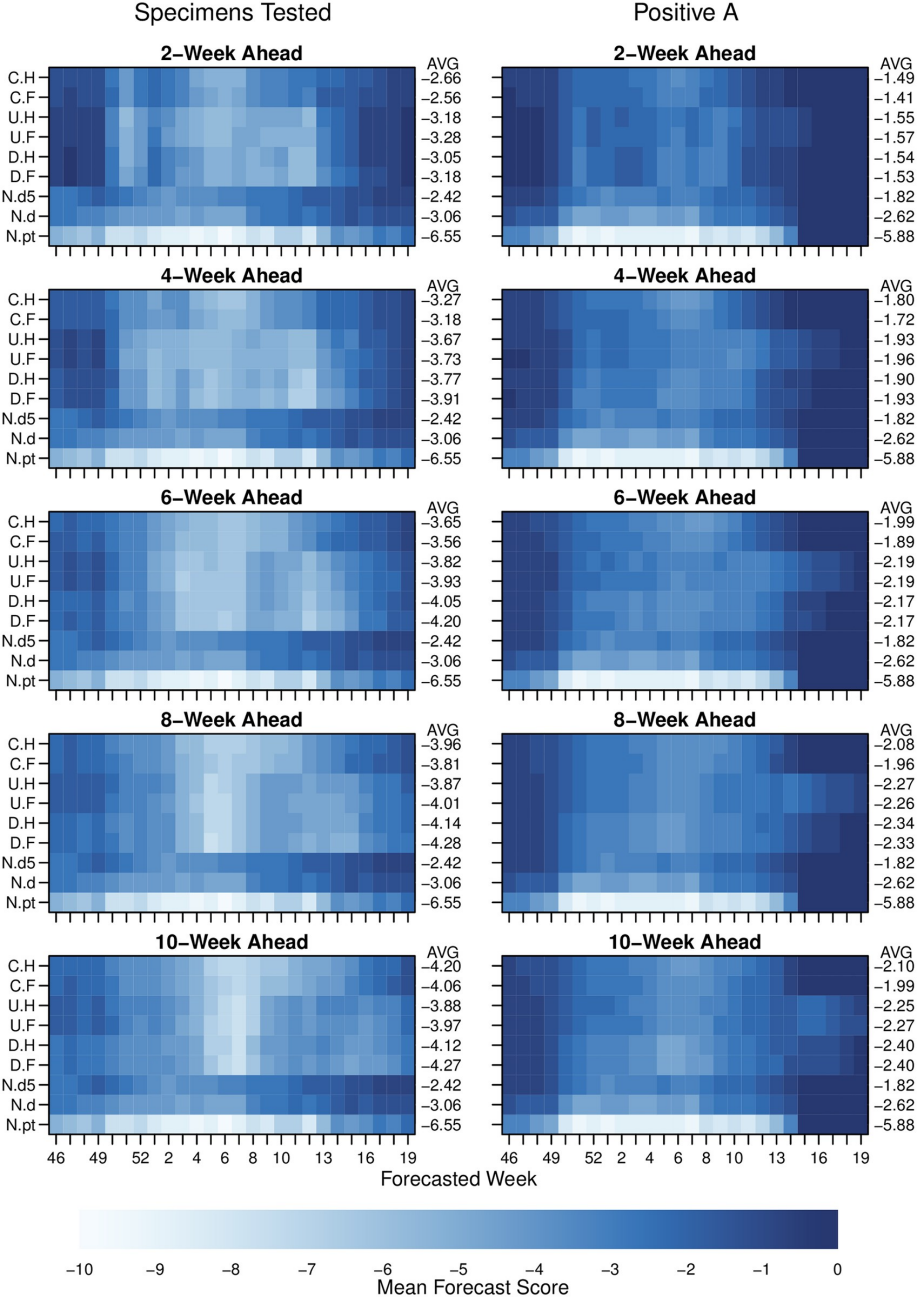
Forecast scores for 2- to 10-weeks ahead lead times, and comparing different data metrics. Pixel colour shows forecast score (see main text) for a given observation week averaged across clusters and seasons, e.g. the 4-week ahead forecast for week 48 was made using data only up to week 44. Averages across all weeks for a given model are printed on the RHS of each row of pixels. Model type is shown on LHS y-axis tick labels: C.H, coupled model with humidity modulated contact rate; C.F, coupled model with fixed contact rate; U.H, uncoupled with humidity; U.F, uncoupled with fixed contact rate; D.H, model directly fitted to cluster with humidity term; D.F, model directly fitted to cluster with fixed contact rate; N.pt, null model made from simple model of that week for all other seasons; N.d, null model made from fitting a log normal to all observations for that week from other years; and N.d5, null model made from fitting log-normal to the observation week, two weeks prior and two weeks following for all other years (see main text). Models are ordered approximately from least complex on the bottom rows to most complex on the top row. See S2 and S3 Figs for complete 1–10 weeks ahead forecast results.

When averaging across all clusters and seasons, at each forecast week; we found substantial evidence that PA data could be more accurately forecast than could TS data (Fig 3 and S3–S5 Figs). We assessed accuracy using a similar scoring system as that used for influenza by the US CDC [8] during the same seasons. The incidence scale was separated into 131 bins and we used our model to assign a probability to every possible category. In the 2019–20 season, the CDC influenza competition moved to a single-bin scoring system. For completeness, we have reproduced S3 and S4 Figs with single-bin scoring as S6 and S7 Figs respectively. The best model for 2-week ahead forecasts scored an average of -1.41 for PA data compared with -2.55 for TS data. Although the accuracy of the forecast did vary over the season, the superiority of best fits to PA data over TS data was maintained across the whole season. In fact, the accuracy of the best fit model for PA data with a 10-week lead time was still better than that of the TS data at a 2-week lead time.

**Fig 4:**
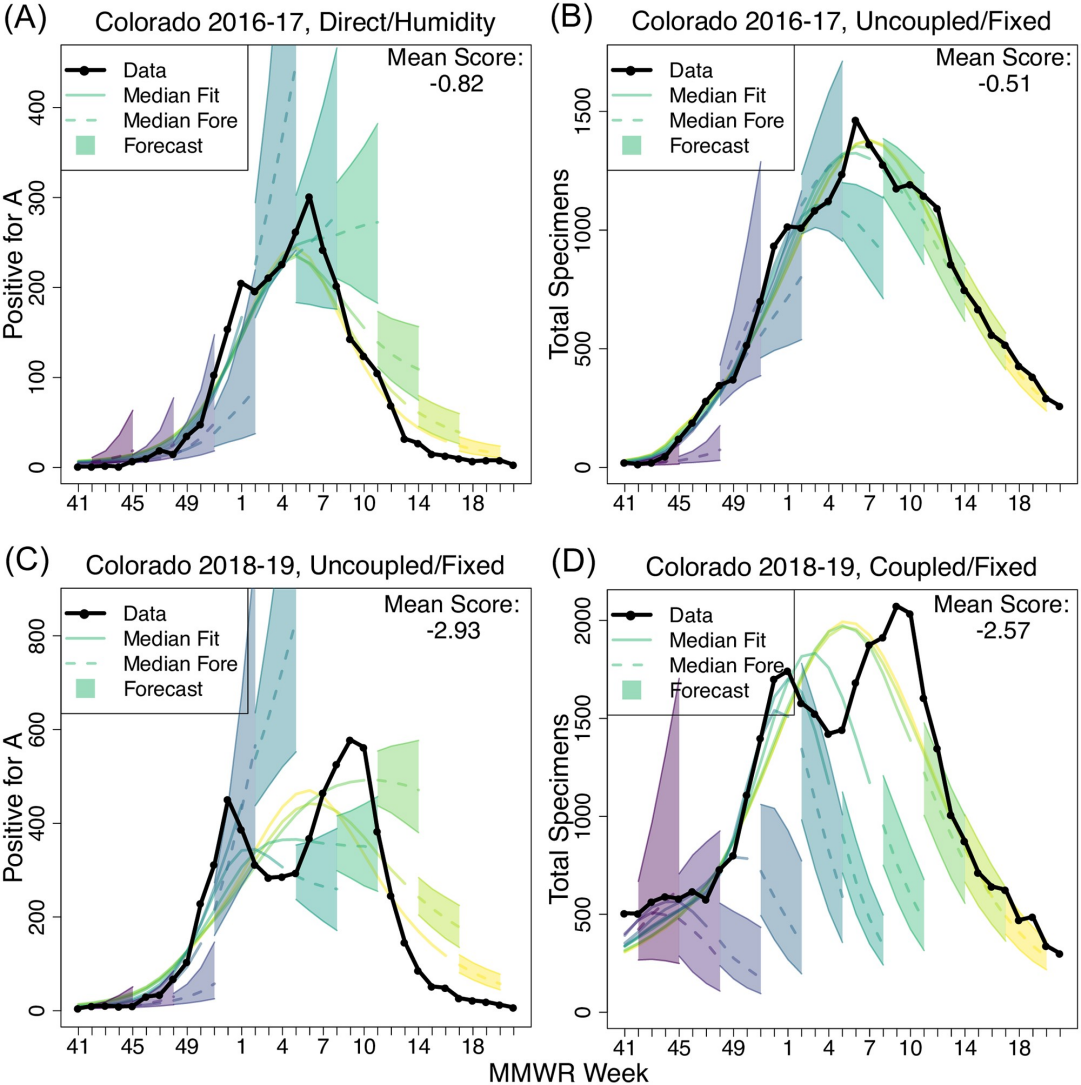
Illustrative high-scoring and lower-scoring forecasts for the Colorado cluster. Here the incidence data is shown in black and shaded cones depict the central 50% forecast windows for 1–4 week ahead. Forecasts were generated for every week, but only every third forecast is shown here for clarity. Colour progression from left to right indicates time of forecast. Solid lines show fit to data at the point the forecast was assumed to have been made. Dashed lines show forecasts beyond the time at which data were assumed to be available. Mean score is the average score across MMWR forecasted weeks and 1 to 4 weeks ahead targets.

We considered specific examples to understand how and why the models were better able to fit these data in different scenarios. For example, after testing all forecast models at all forecast times for the Colorado cluster for 2016–17, the best fitting model was better able to capture the rapid acceleration of incidence and the shape of the peak PA data stream than was possible for the best fitting model for the TS data (Fig 4A and 4B). Despite not being able to accurately fit the decay phase of the epidemic, the best fitting model for the PA data still achieved a substantially better score of -0.51 than the score of -0.82 that was possible for the corresponding TS data. We note that a score of -0.51 corresponds to 60% of forecasts falling within the skill window. However, for the same cluster for the following year, the same did not apply. The best model fits to both datasets were substantially worse than the year before, largely due to the cluster exhibiting a double peak (Fig 4C and 4D). The average single-peak line through the slightly diffuse TS data performed slightly better than did a similar line through the more sharply peaked PA data. We note that our data and models do not distinguish between subtypes of influenza A and that both H1N1 and H3N2 were circulating during similar periods in this season. Similar panels are available for all models and seasons in S12 Fig. Looking at the seasons more broadly, S8 Fig contains scores averaged over cluster and 1- to 4-weeks-ahead forecasts to show model performance by season and data type. Here we see relatively high scores for the 2016–17 season, likely a result of semi-synchronized H3N2 and B peaks. The 2017–18 season was characterized by an early H3N2 peak followed by a late B peak. Thus, the model performed well on PA data, but struggled with the broad peaks in TS data. A--very unusual--early H1N1 peak followed by late H3N2 peak caused 2018–19 to score the worst for both TS and PA data.

Among the mechanistic models, coupled models generally performed better than uncoupled models or models that fit the cluster incidence directly (Fig 3). Also, the accuracy of mechanistic models relative to non-mechanistic average-based models compared for TS and PA data suggests that mechanistic models are able to capture more information from the PA data than a simple baseline (null) model. For the TS data, the 5-week rolling historical average model outperformed the best mechanistic models for all time scales from 2- to 10-weeks. However, for PA data, the best mechanistic model outperformed the 5-week average historical model in the 1- to 4-week ahead range. We note that there were few years available to the historical models in these data, therefore the performance of historical-average models would likely improve over time as the training data set grows.

**Fig 5:**
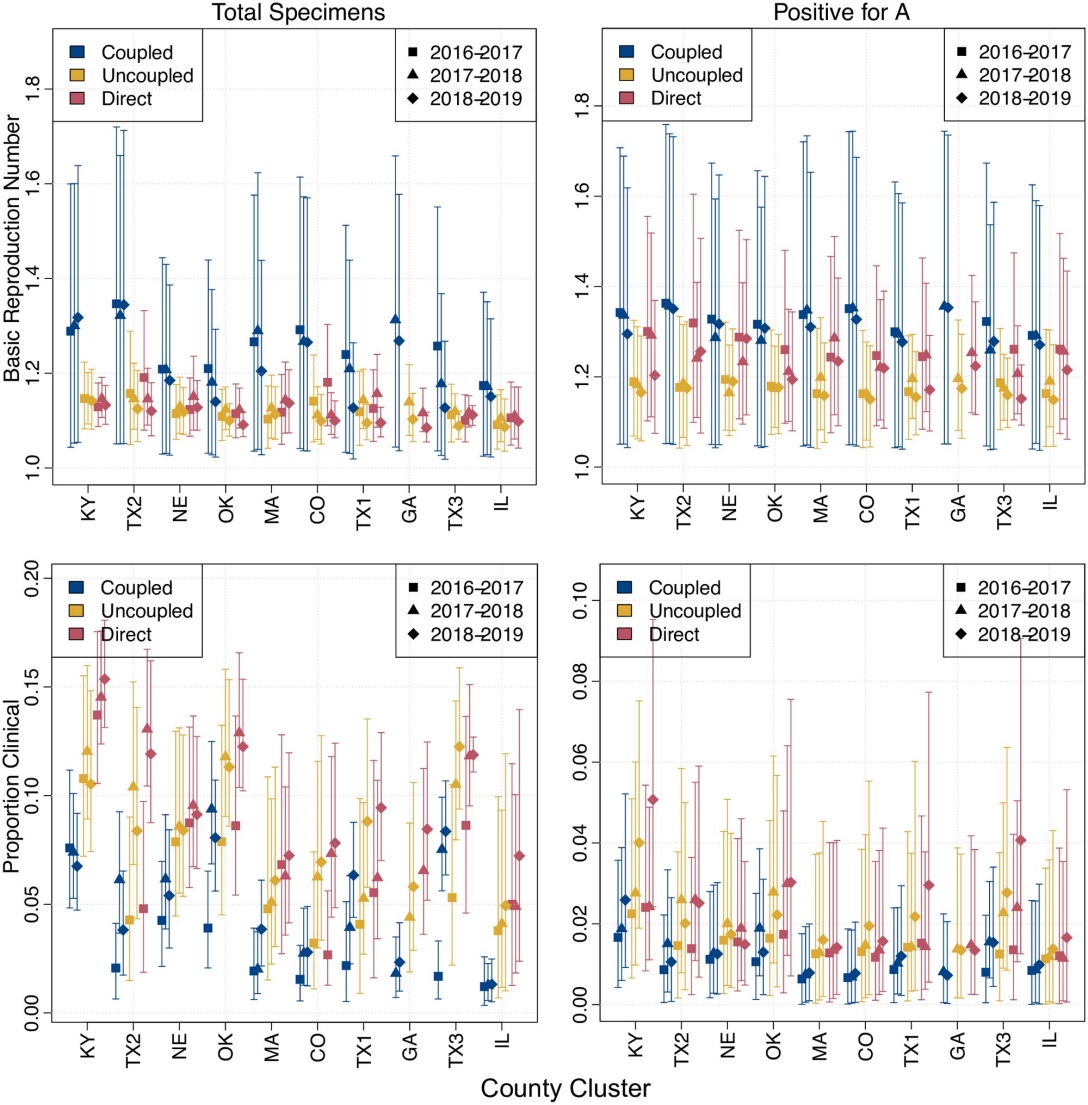
Posterior parameter densities by season, cluster and model type. Bars show central 95% of posterior credibility interval with marker showing posterior mean. Colour indicates model type and symbol shape indicates season. Values for each cluster are grouped together as per x-axis tick labels.

Patterns in the posterior densities for key mechanistic parameters also suggest that when the models are fit to the PA data they are capturing more of the underlying biology (Fig 5). The posterior median for the basic reproductive number *R*_0_ was consistent from year to year and location to location when fit to the PA data. For coupled models, cluster *R*_0_s were higher (mean 1.32; 95% CI [1.31, 1.33]) than for uncoupled models (mean 1.18; 95% CI [1.17, 1.18]). Given that transmissibility is reflected by *R*_0_ − 1, this difference is significant and likely reflects the need for uncoupled models to have longer durations (resulting from lower *R*_0_) because the overall cluster incidence was not driven by epidemics in subpopulations taking off at different times. This theory is supported by an inverse relationship for the estimated proportion of infections which result in clinical cases *p*_*C*_. Estimates of *p*_*C*_ were lower for coupled models than for uncoupled models because subpopulations with higher *R*_0_s require fewer of their infections to result in cases to achieve the same level of incidence as those from the uncoupled model. Despite the coupled model outperforming the uncoupled model, estimates for the parameters that determined coupling strength were not well constrained when fit to the data (S9 Fig). Although coupling posteriors showed large spreads, there were subtle differences from cluster to cluster and between the data metrics. Plotting the posterior coupling kernel *κ* as a function of distance (see S10 and S11 Figs) shows differing patterns of coupling fall-off.

## Discussion

We have shown for a large set of mechanistic models, using a skill scoring system analogous to current best practise, that cluster-level influenza incidence-data based on type-confirmed test results generate substantially better forecasts than those based only on the volume of respiratory samples being tested. We have also shown a close correlation between the weekly total number of respiratory samples in each cluster and the pattern of influenza-like illness reported to the national surveillance system for the geographical region enclosing each cluster. Among these mechanistic models, cluster forecasts were more accurate in those that fit to subpopulation county data than those that do not. Also, the relative difference between the mechanistic models and simple historical average models is much greater when forecasting type-specific data than when forecasting total samples.

A limitation of our study is that we compared only forecasts from our suite of mechanistic and historical average models against the two datasets. Therefore, it is possible that other approaches may have been able to forecast the TS data more effectively than the PA data. However, the difference in our ability to forecast the two types of data was considerable and significantly greater than the difference in performance between our approach and other high performing participants in the CDC challenge study, which is measured on a similar scale [8]. Also, intuitively, the shape of epidemics in the PA data was more consistent with a short-generation-time infectious disease epidemic: the degree of noise prior to the start of the season was low and the epidemic itself was more peaked, as measured by width at half maximum, suggesting that the substantial improvement in forecastability using a mechanistic approach is consistent with the PA data being much more representative of the underlying biological process.

A previous study compared the forecastability of pathogen-specific data and found evidence of slightly increased forecastability of local type- and subtype-specific data, albeit using different accuracy metrics [28]. However, the virological data available to the prior study represented considerably larger spatial units than the separate ILI data. More local sub-type epidemics were inferred by combining regional laboratory-confirmed positive proportions with Google Flu Trends [50] estimates of state and municipality ILI data, implicitly assuming that the temporal distribution of types and subtypes was uniform for large geographical regions. A similar study [29] looked at influenza positive laboratory data at small spatial scale combined with several other data types, but did not address subpopulation coupling or type-specific data. We suggest that aggregated positive tests at the geographical and temporal scales of analysis contain maximal information that is not present in similar inferred data streams.

Our results suggest a number of additional refinements to our forecasting workflow that may further improve model forecast accuracy. We assumed that each cluster consisted only of the sub-populations for which data were available. It may be that the inclusion of the intervening and surrounding populations that were not observed could increase accuracy. This would substantially increase the computational complexity of the approach, but could potentially fill-in surveillance gaps at the same time as increasing the accuracy and robustness of the forecasts. Additionally, tightly clustered counties with low reporting could be combined into a single super-county. This would allow us to include incidence data that is currently being lost in the county filtering process. A separate issue is that we have treated these as weekly data, which reflects their current reporting pattern. However, accuracy for now-casting and short term forecasting could be improved substantially by looking at daily incidence which are available from this surveillance network. Although some work would be required to make a day-of-week adjustment [51], accuracy of 1-week ahead and 2-week ahead forecasts would likely increase substantially over and above the gains described here.

Public health authorities may wish to consider the routine use of type- and subtype-specific point-of-care data for influenza surveillance in addition to traditional ILI surveillance to enable more accurate local influenza forecasts during seasonal and pandemic periods. Our results were for 10 geographical clusters based only on three seasons of data (so a maximum of two training years). Very soon, the network of machines from which these data were obtained and other similar networks will have much more complete geographical and temporal coverage [52]. Similar networks are being established in many other populations.

Other studies have shown that aggregating local forecasts can improve accuracy over direct forecasts for larger geographical units, but have not focused on the potential accuracy at small spatial scales [11, 53]. Also, more descriptive ecological analyses have demonstrated the potential for improved local forecasts by characterizing how humidity and population density are correlated with average properties of epidemic curves [54]. Building on these prior studies, the smaller spatial unit and temporal accuracy of the results presented here could be used to guide public health interventions and give individuals the opportunity to modify their behaviour over short periods of time, based on very local information. In particular, people at high risk of severe disease may choose to reduce their social mixing during short periods where the local risk of infection can be accurately predicted to be high.

## Supporting information

S1 Fig County filtering by season. Counties with at least 250 specimens and 40 reporting weeks per season. (A) 2016–17 season—187 counties, (B) 2017–18 season—281 counties, and (C) 2018–19 season—541 counties. The map base layer was sourced from the U.S. Census Bureau [39]
(PDF)

S2 Fig Peak-normalization of scoring bins across data metrics, illustrated for 2016–2017 season in the San Antonio / Austin area cluster. Bins were scaled to each metric such that all peaks fall in the same bin. The skillful bins for the peak week are shown in yellow shading. Forecast probabilities in these bins, in addition to the observed bin (red shading), are summed to calculate skill. The Centers for Disease Control and Prevention percentage ILI (CDC %ILI) is for the enclosing CDC region (state of Texas).
(PDF)

S3 Fig Mean scores for total specimens forecasts 1–10 weeks ahead. Pixel colour shows forecast score (see main text) for a given observation week averaged across clusters and seasons, e.g. the 4-week ahead forecast for week 48 was made using data only up to week 44. Averages across all weeks for a given model are printed on the RHS of each row of pixels. Model type is shown on LHS y-axis tick labels: C.H, coupled model with humidity modulated contact rate; C.F, coupled model with fixed contact rate; U.H, uncoupled with humidity; U.F, uncoupled with fixed contact rate; D.H, model directly fitted to cluster with humidity term; D.F, model directly fitted to cluster with fixed contact rate; N.pt, null model made from simple model of that week for all other seasons; N.d, null model made from fitting a log normal to all observations for that week from other years; and N.d5, null model made from fitting log-normal to the observation week, two weeks prior and two weeks following for all other years (see main text). Models are ordered approximately from least complex on the bottom rows to most complex on the top row.
(PDF)

S4 Fig Mean scores for positive-for-A forecasts 1–10 weeks ahead. Pixel colour shows forecast score (see main text) for a given observation week averaged across clusters and seasons, e.g. the 4-week ahead forecast for week 48 was made using data only up to week 44. Averages across all weeks for a given model are printed on the RHS of each row of pixels. Model type is shown on LHS y-axis. Models are ordered approximately from least complex on the bottom rows to most complex on the top row.
(PDF)

S5 Fig Peak target mean forecast scores for total specimens and positive for A. The panels are divided by target (top—Peak Week, bottom—Peak Intensity) and data metric (left—total specimens tested, right—specimens positive for A). Forecast scores for each square are averaged across all clusters and all seasons. The mean value of each row appears in the AVG column on the right. Model type is shown on LHS y-axis.
(PDF)

S6 Fig Mean single-bin scores for total specimens forecasts 1–10 weeks ahead. Pixel colour shows forecast single-bin score for a given observation week averaged across clusters and seasons, e.g. the 4-week ahead forecast for week 48 was made using data only up to week 44. Averages across all weeks for a given model are printed on the RHS of each row of pixels. Model type is shown on LHS y-axis.
(PDF)

S7 Fig Mean single-bin scores for positive-for-A forecasts 1–10 weeks ahead. Pixel colour shows forecast single-bin score for a given observation week averaged across clusters and seasons, e.g. the 4-week ahead forecast for week 48 was made using data only up to week 44. Averages across all weeks for a given model are printed on the RHS of each row of pixels. Model type is shown on LHS y-axis.
(PDF)

S8 Fig Forecast scores for 1- to 4-week-ahead targets by season. Pixel color shows multi-bin forecast score (see main text) for a given observation week averaged across clusters and 1–4 week ahead target. Averages across all weeks of the season for a given model are printed on the RHS of each row of pixels. Model type is shown on LHS y-axis.
(PDF)

S9 Fig Coupling parameter mean and central 80% interval. Results were compiled from MCMC chains across all models and forecast weeks for a given cluster and season. Coupling is modeled with the offset power law relationship described in (11) using free parameters for saturation distance and power. Briefly, the probability of coupling occurring across space is determined by a function with a plateau followed by a power law drop-off. Saturation distance is the width of the plateau and ‘power’ is the power coefficient of the drop-off.
(PDF)

S10 Fig Spatial kernel posterior by cluster for positive-for-A. Spatial kernel *κ* posterior as a function of distance. Posterior distributions are across all season-years and *β*-modulation variants (fixed, humidity) for each cluster. Posteriors are taken from late-season fits using data through EW 18. Median kernel values appear as a black line and the 25% to 75% interval is shaded blue. For each unique county pair, there appears a vertical orange line indicating the great-circle distance (km) between population centroids.
(PDF)

S11 Fig Spatial kernel posterior by cluster for total specimens. Spatial kernel *κ* posterior as a function of distance. Posterior distributions are across all season-years and *β*-modulation variants (fixed, humidity) for each cluster. Posteriors are taken from late-season fits using data through EW 18. Median kernel values appear as a black line and the 25% to 75% interval is shaded blue. For each unique county pair, there appears a vertical orange line indicating the great-circle distance (km) between population centroids.
(PDF)

S12 Fig Illustrative forecasts for all models. Each page contains results from a single model. Rows are divided first by data metric and then by season. Columns are divided by cluster. In the panels incidence data is shown in black and shaded cones depict the central 50% forecast windows for 1–4 week ahead. Forecasts were generated for every week, but only every third forecast is shown here for clarity. Colour progression from left to right indicates time of forecast. Solid lines show median fit to data at the point the forecast was assumed to have been made. Dashed lines show forecasts beyond the time at which data were assumed to be available. Mean score is the average score across MMWR forecasted weeks and 1 to 4 weeks ahead targets.
(PDF)

S1 Table Pearson correlations between data metrics. Correlations are calculated for each cluster and season, and comparing total specimens, positive for, A and CDC %ILI. From the resulting distributions, we have extracted the median, 20%, and 80% quantiles.
(PDF)

S2 Table County cluster information. (PNG)
