# Supplementary figures and images for: Accurate influenza forecasts using type-specific incidence data for small geographic units
Source: PLoS Comput Biol. 2021 Jul 29;17(7):e1009230. doi: 10.1371/journal.pcbi.1009230 (PMC8354478; doi:10.1371/journal.pcbi.1009230)

A.

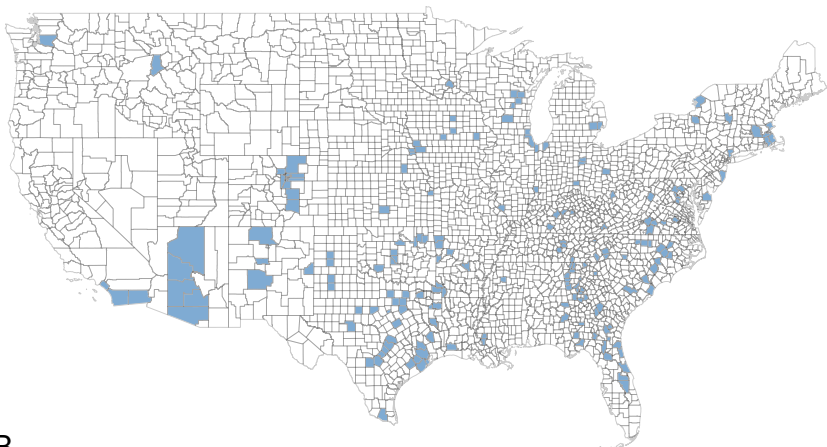

B.

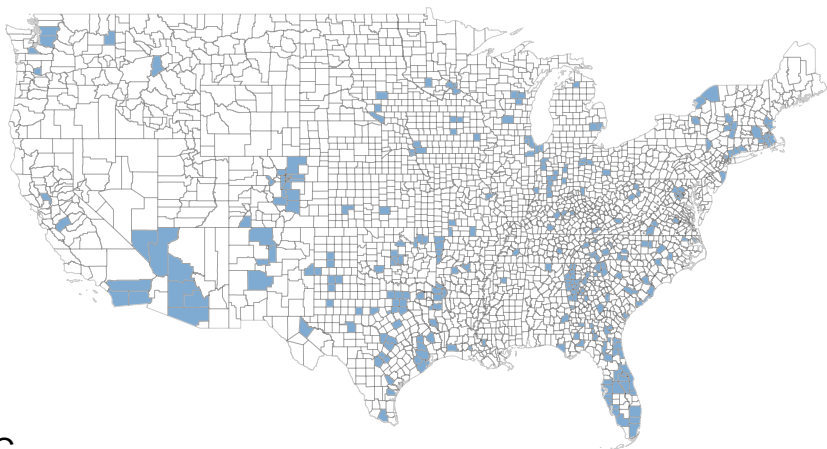

C.

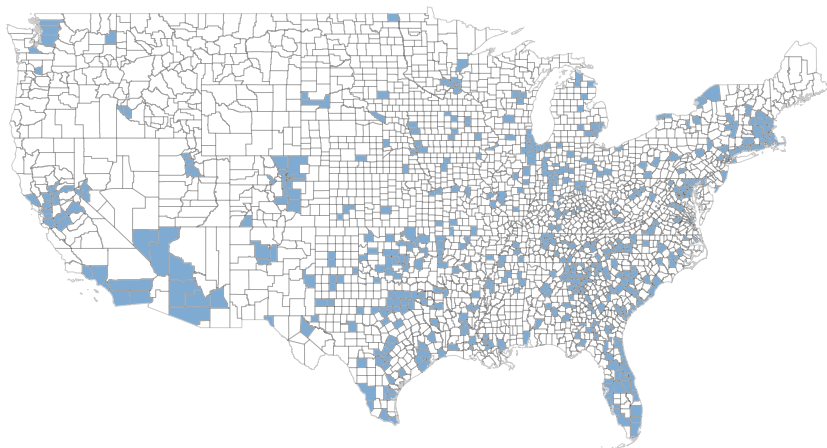

Supplement: S1 Fig — Counties with at least 250 specimens and 40 reporting weeks per season. (A) 2016–17 season—187 counties, (B) 2017–18 season—281 counties, and (C) 2018–19 season—541 counties. The map base layer was sourced from the U.S. Census Bureau [39] (PDF) [file pcbi.1009230.s001.pdf]

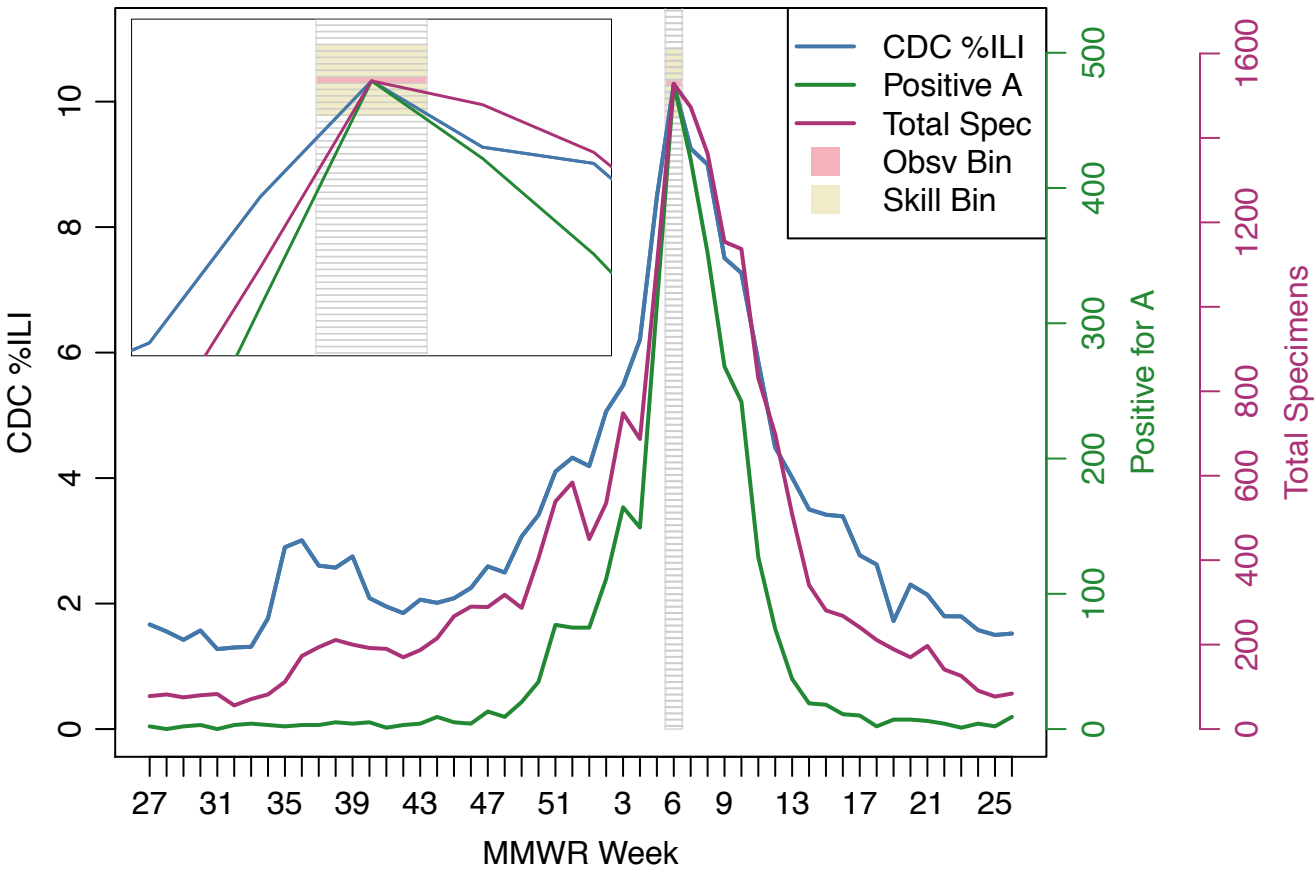

Supplement: S2 Fig — Bins were scaled to each metric such that all peaks fall in the same bin. The skillful bins for the peak week are shown in yellow shading. Forecast probabilities in these bins, in addition to the observed bin (red shading), are summed to calculate skill. The Centers for Disease Control and Prevention percentage ILI (CDC %ILI) is for the enclosing CDC region (state of Texas). (PDF) [file pcbi.1009230.s002.pdf]

## Specimens Tested

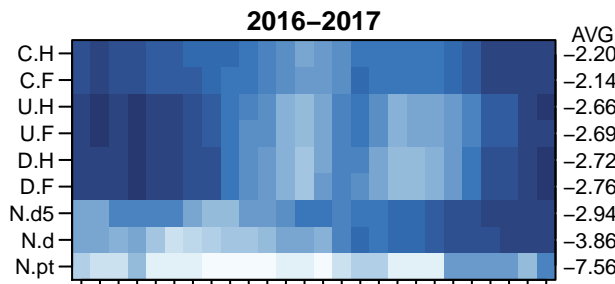

## Positive A

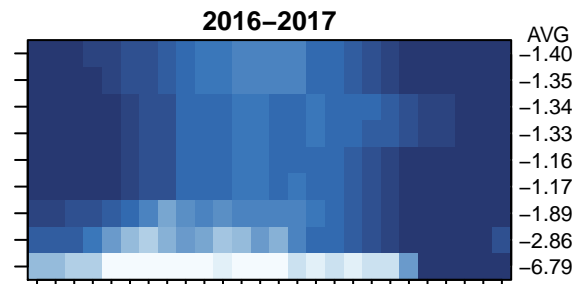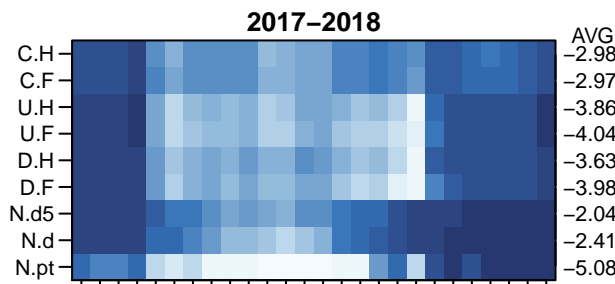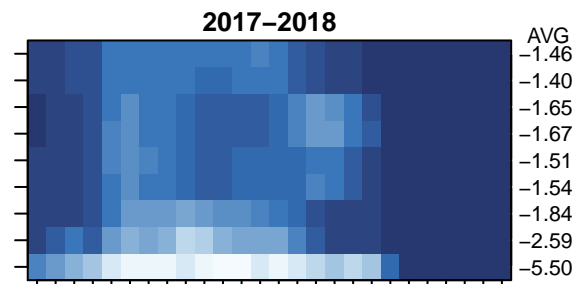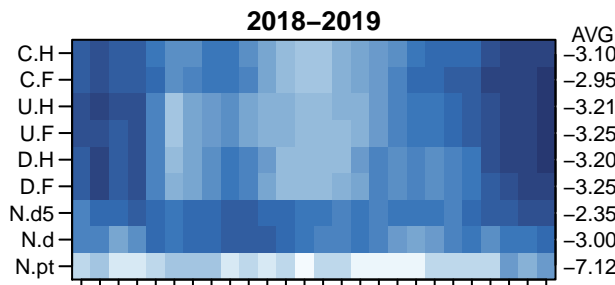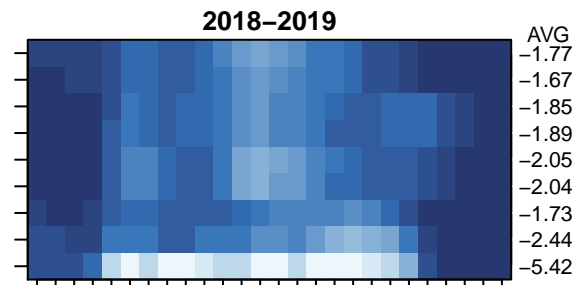

46 49 52 2 4 6 8 10 13 16 19

46 49 52 2 4 6 8 10 13 16 19

Forecasted Week

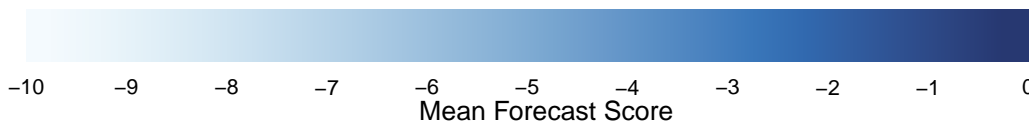

Supplement: S8 Fig — Pixel color shows multi-bin forecast score (see main text) for a given observation week averaged across clusters and 1–4 week ahead target. Averages across all weeks of the season for a given model are printed on the RHS of each row of pixels. Model type is shown on LHS y-axis. (PDF) [file pcbi.1009230.s008.pdf]

Total Specimens

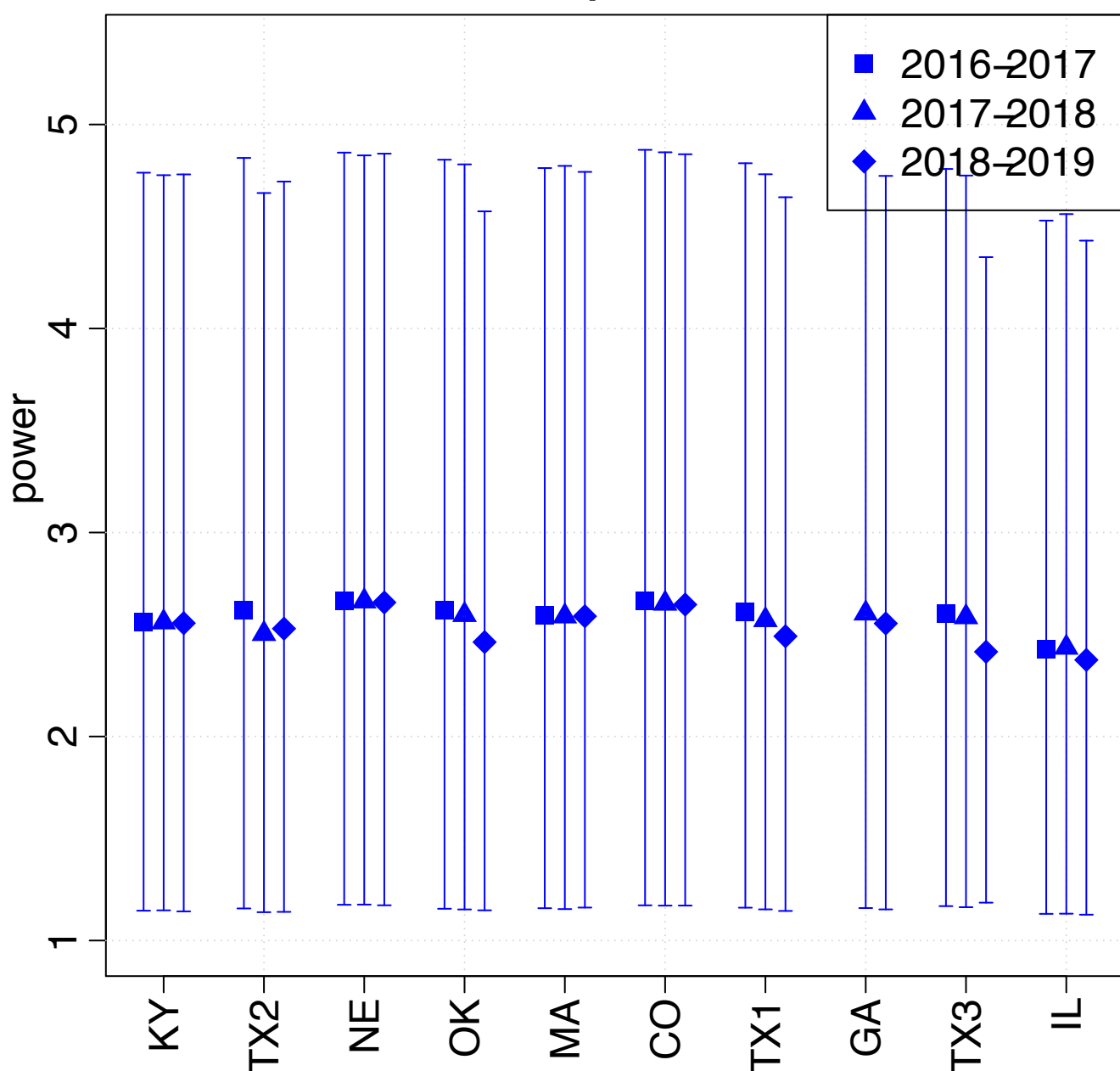

Positive for A

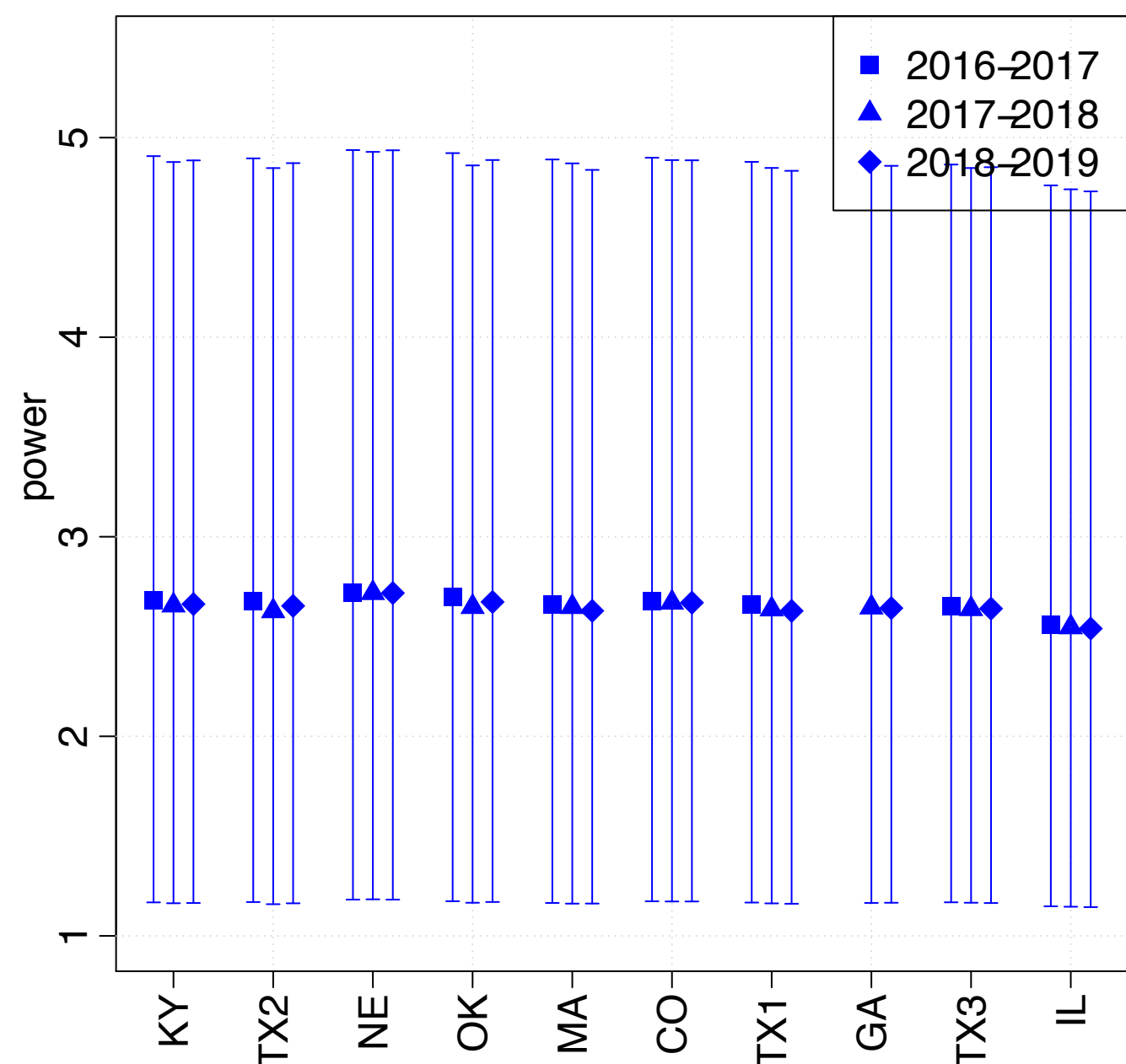

Total Specimens

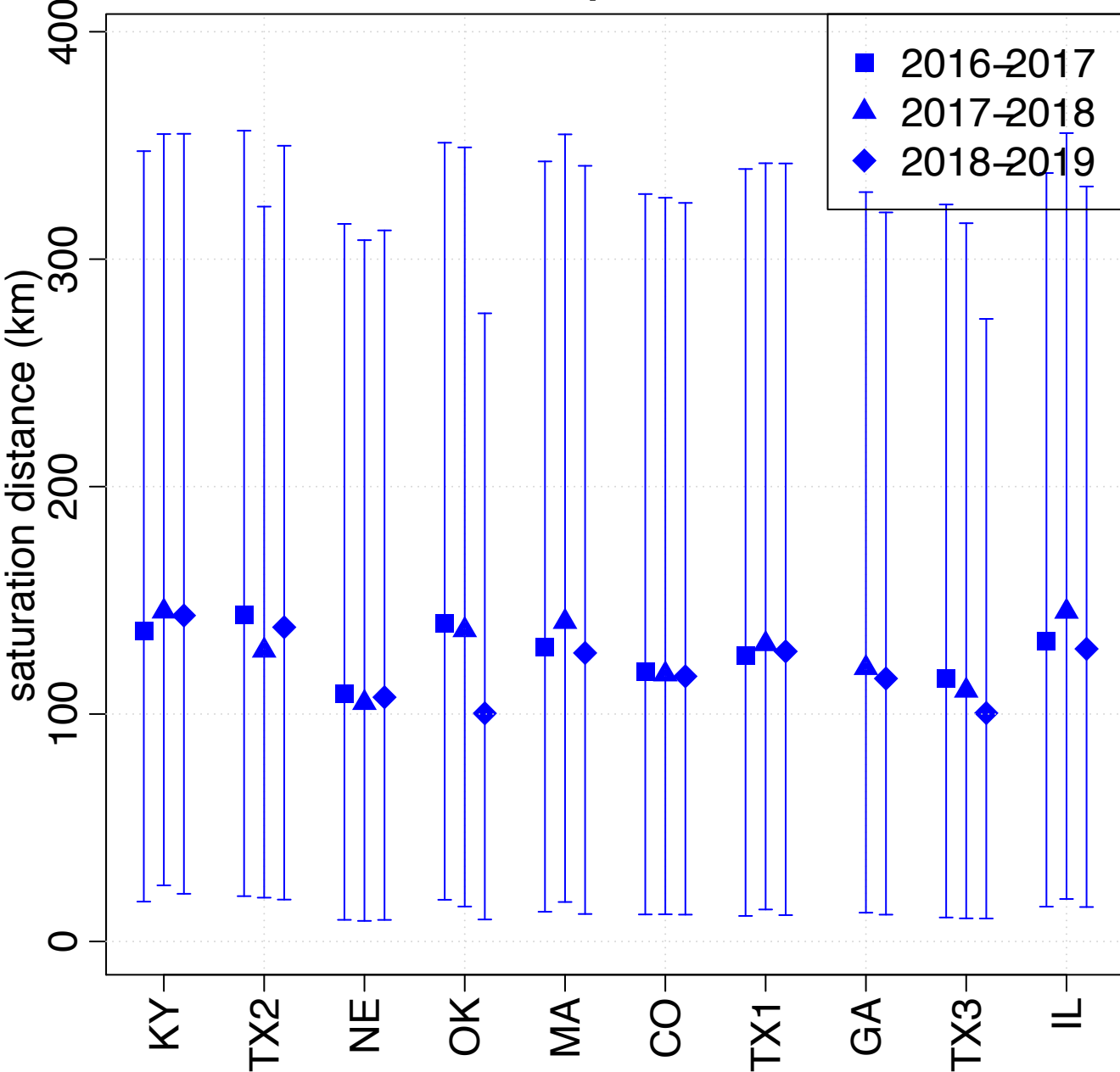

Positive for A

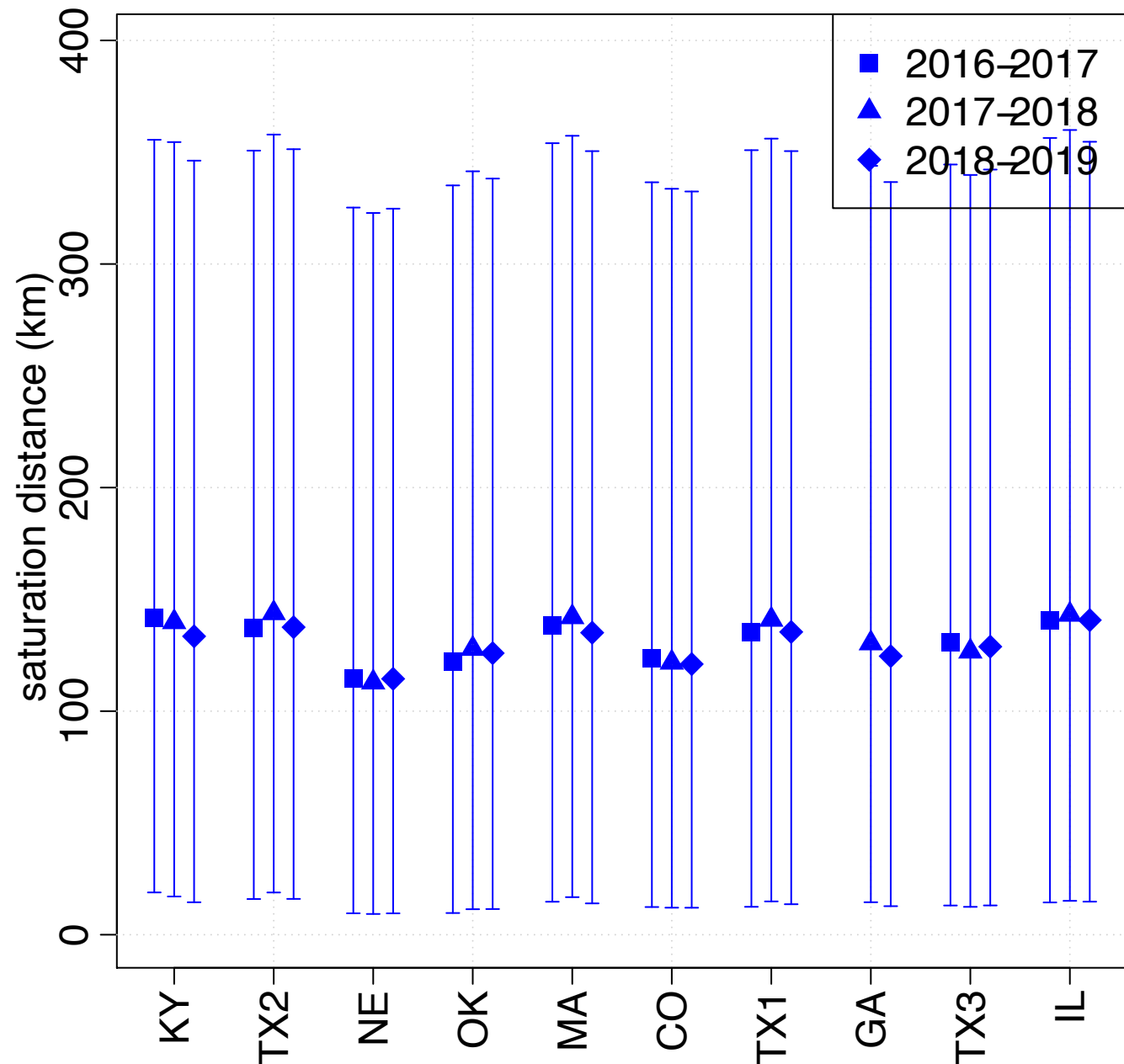

Supplement: S9 Fig — Results were compiled from MCMC chains across all models and forecast weeks for a given cluster and season. Coupling is modeled with the offset power law relationship described in (11) using free parameters for saturation distance and power. Briefly, the probability of coupling occurring across space is determined by a function with a plateau followed by a power law drop-off. Saturation distance is the width of the plateau and ‘power’ is the power coefficient of the drop-off. (PDF) [file pcbi.1009230.s009.pdf]

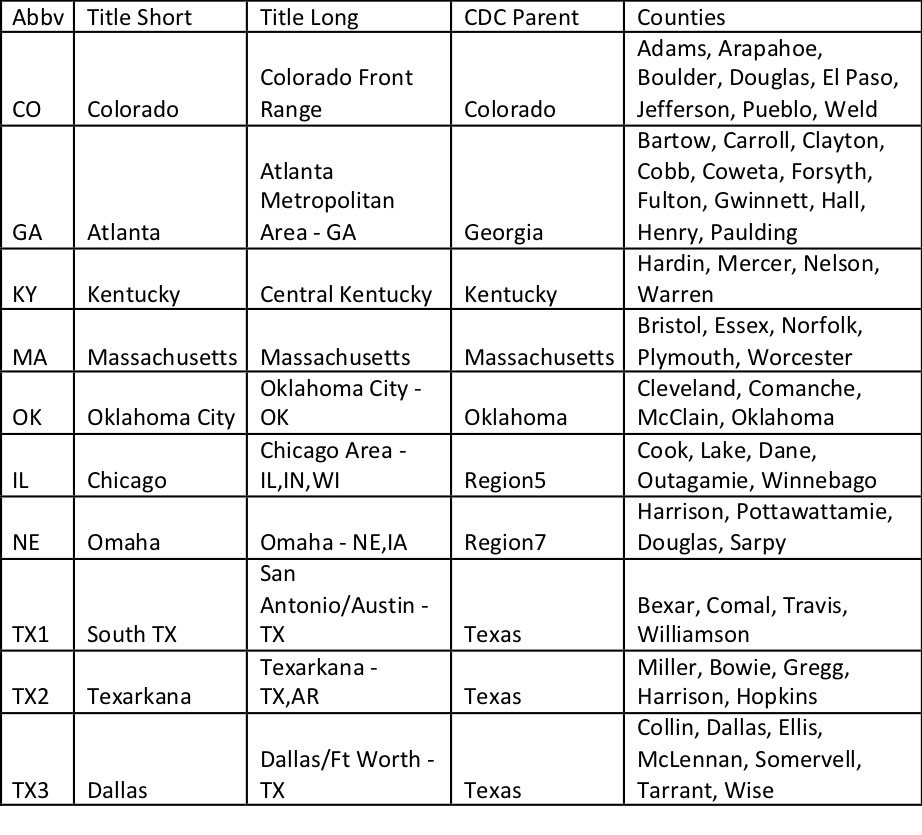

Supplement: S2 Table — (PNG) [file pcbi.1009230.s014.png]
