# Supplementary material for: Accurate influenza forecasts using type-specific incidence data for small geographic units
Source: PLoS Comput Biol. 2021 Jul 29;17(7):e1009230. doi: 10.1371/journal.pcbi.1009230 (PMC8354478; doi:10.1371/journal.pcbi.1009230)
Supplement: S3 Fig — Pixel colour shows forecast score (see main text) for a given observation week averaged across clusters and seasons, e.g. the 4-week ahead forecast for week 48 was made using data only up to week 44. Averages across all weeks for a given model are printed on the RHS of each row of pixels. Model type is shown on LHS y-axis tick labels: C.H, coupled model with humidity modulated contact rate; C.F, coupled model with fixed contact rate; U.H, uncoupled with humidity; U.F, uncoupled with fixed contact rate; D.H, model directly fitted to cluster with humidity term; D.F, model directly fitted to cluster with fixed contact rate; N.pt, null model made from simple model of that week for all other seasons; N.d, null model made from fitting a log normal to all observations for that week from other years; and N.d5, null model made from fitting log-normal to the observation week, two weeks prior and two weeks following for all other years (see main text). Models are ordered approximately from least complex on the bottom rows to most complex on the top row. (PDF) [file pcbi.1009230.s003.pdf]

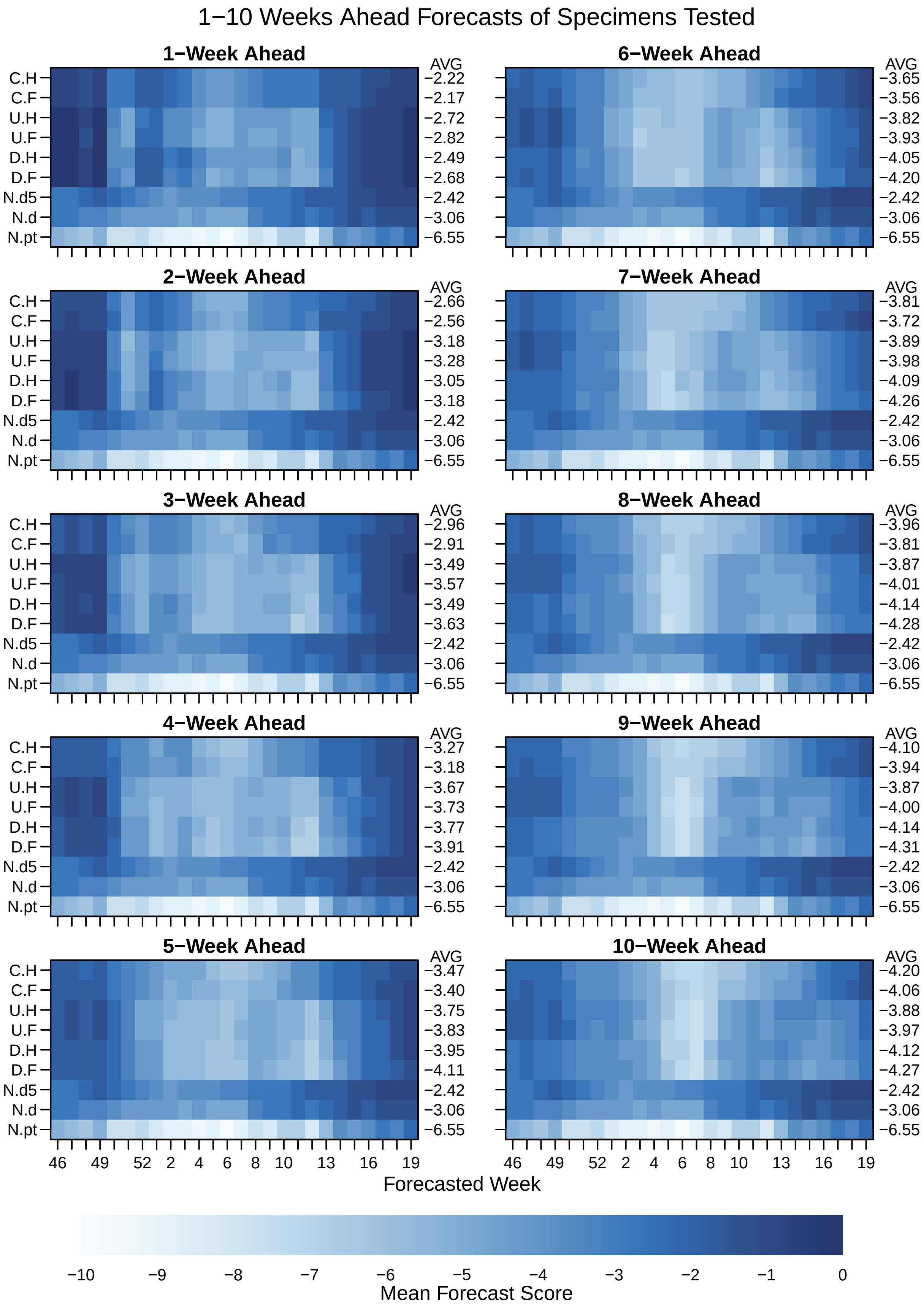

1−10 Weeks Ahead Forecasts of Specimens Tested
1−Week Ahead
AVG
C.H −2.22
C.F −2.17
U.H −2.72
U.F −2.82
D.H −2.49
D.F −2.68
N.d5 −2.42
N.d −3.06
N.pt −6.55
2−Week Ahead
C.H −2.66
C.F −2.56
U.H −3.18
U.F −3.28
D.H −3.05
D.F −3.18
N.d5 −2.42
N.d −3.06
N.pt −6.55
3−Week Ahead
C.H −2.96
C.F −2.91
U.H −3.49
U.F −3.57
D.H −3.49
D.F −3.63
N.d5 −2.42
N.d −3.06
N.pt −6.55
4−Week Ahead
C.H −3.27
C.F −3.18
U.H −3.67
U.F −3.73
D.H −3.77
D.F −3.91
N.d5 −2.42
N.d −3.06
N.pt −6.55
5−Week Ahead
C.H −3.47
C.F −3.40
U.H −3.75
U.F −3.83
D.H −3.95
D.F −4.11
N.d5 −2.42
N.d −3.06
N.pt −6.55
6−Week Ahead
−3.65
−3.56
−3.82
−3.93
−4.05
−4.20
−2.42
−3.06
−6.55
7−Week Ahead
−3.81
−3.72
−3.89
−3.98
−4.09
−4.26
−2.42
−3.06
−6.55
8−Week Ahead
−3.96
−3.81
−3.87
−4.01
−4.14
−4.28
−2.42
−3.06
−6.55
9−Week Ahead
−4.10
−3.94
−3.87
−4.00
−4.14
−4.31
−2.42
−3.06
−6.55
10−Week Ahead
−4.20
−4.06
−3.88
−3.97
−4.12
−4.27
−2.42
−3.06
−6.55
46 49 52 2 4 6 8 10 13 16 19
Forecasted Week
−10 −9 −8 −7 −6 −5 −4 −3 −2 −1 0
Mean Forecast Score
