# Supplementary material for: Accurate influenza forecasts using type-specific incidence data for small geographic units
Source: PLoS Comput Biol. 2021 Jul 29;17(7):e1009230. doi: 10.1371/journal.pcbi.1009230 (PMC8354478; doi:10.1371/journal.pcbi.1009230)
Supplement: S4 Fig — Pixel colour shows forecast score (see main text) for a given observation week averaged across clusters and seasons, e.g. the 4-week ahead forecast for week 48 was made using data only up to week 44. Averages across all weeks for a given model are printed on the RHS of each row of pixels. Model type is shown on LHS y-axis. Models are ordered approximately from least complex on the bottom rows to most complex on the top row. (PDF) [file pcbi.1009230.s004.pdf]

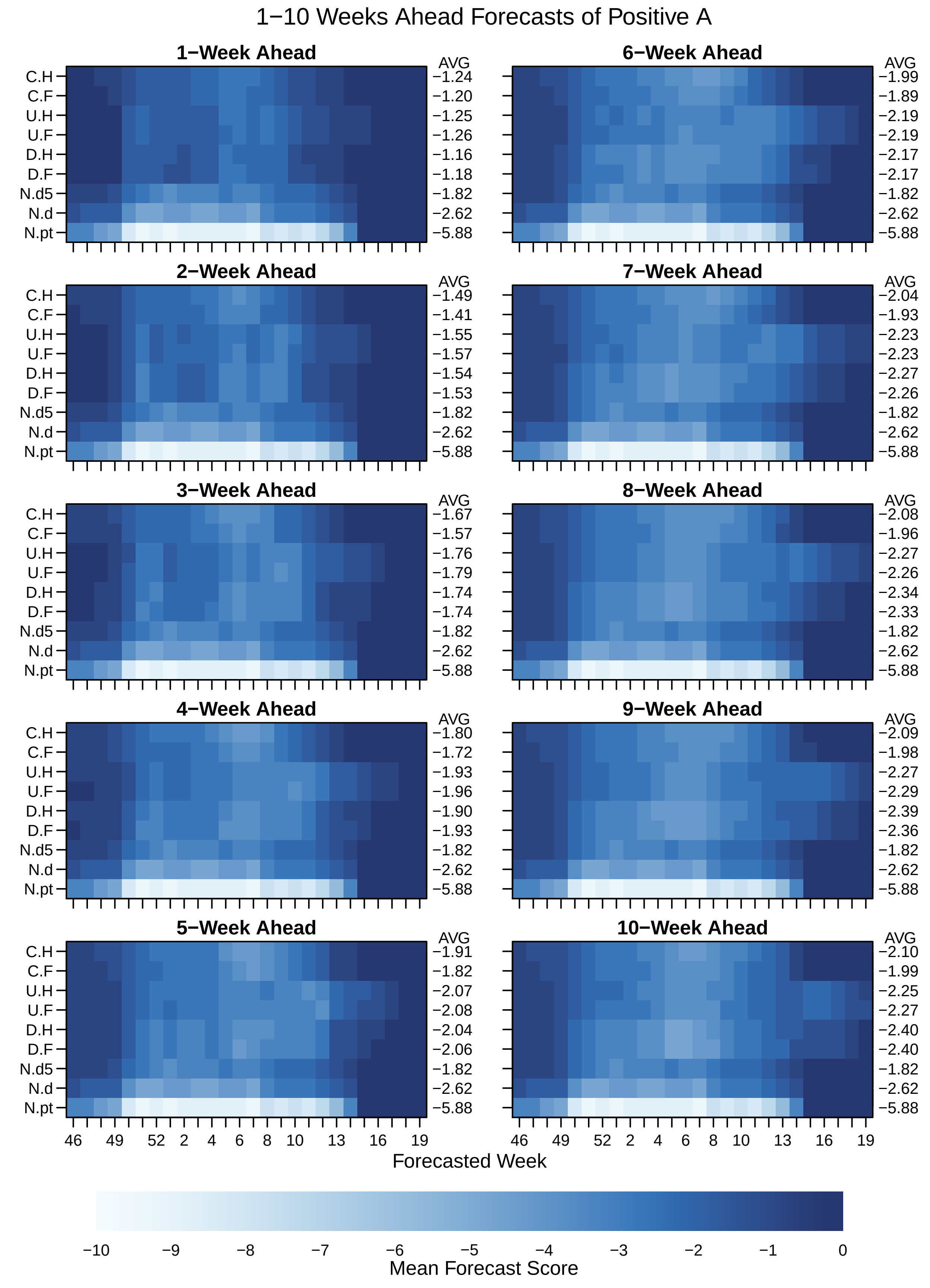
1−10 Weeks Ahead Forecasts of Positive A
1−Week Ahead
C.H C.F U.H U.F D.H D.F N.d5 N.d N.pt
AVG −1.24 −1.20 −1.25 −1.26 −1.16 −1.18 −1.82 −2.62 −5.88
2−Week Ahead
AVG −1.49 −1.41 −1.55 −1.57 −1.54 −1.53 −1.82 −2.62 −5.88
3−Week Ahead
AVG −1.67 −1.57 −1.76 −1.79 −1.74 −1.74 −1.82 −2.62 −5.88
4−Week Ahead
AVG −1.80 −1.72 −1.93 −1.96 −1.90 −1.93 −1.82 −2.62 −5.88
5−Week Ahead
AVG −1.91 −1.82 −2.07 −2.08 −2.04 −2.06 −1.82 −2.62 −5.88
6−Week Ahead
AVG −1.99 −1.89 −2.19 −2.19 −2.17 −2.17 −1.82 −2.62 −5.88
7−Week Ahead
AVG −2.04 −1.93 −2.23 −2.23 −2.27 −2.26 −1.82 −2.62 −5.88
8−Week Ahead
AVG −2.08 −1.96 −2.27 −2.26 −2.34 −2.33 −1.82 −2.62 −5.88
9−Week Ahead
AVG −2.09 −1.98 −2.27 −2.29 −2.39 −2.36 −1.82 −2.62 −5.88
10−Week Ahead
AVG −2.10 −1.99 −2.25 −2.27 −2.40 −2.40 −1.82 −2.62 −5.88
46 49 52 2 4 6 8 10 13 16 19
Forecasted Week
−10 −9 −8 −7 −6 −5 −4 −3 −2 −1 0
Mean Forecast Score
