# Supplementary material for: Accurate influenza forecasts using type-specific incidence data for small geographic units
Source: PLoS Comput Biol. 2021 Jul 29;17(7):e1009230. doi: 10.1371/journal.pcbi.1009230 (PMC8354478; doi:10.1371/journal.pcbi.1009230)
Supplement: S5 Fig — The panels are divided by target (top—Peak Week, bottom—Peak Intensity) and data metric (left—total specimens tested, right—specimens positive for A). Forecast scores for each square are averaged across all clusters and all seasons. The mean value of each row appears in the AVG column on the right. Model type is shown on LHS y-axis. (PDF) [file pcbi.1009230.s005.pdf]

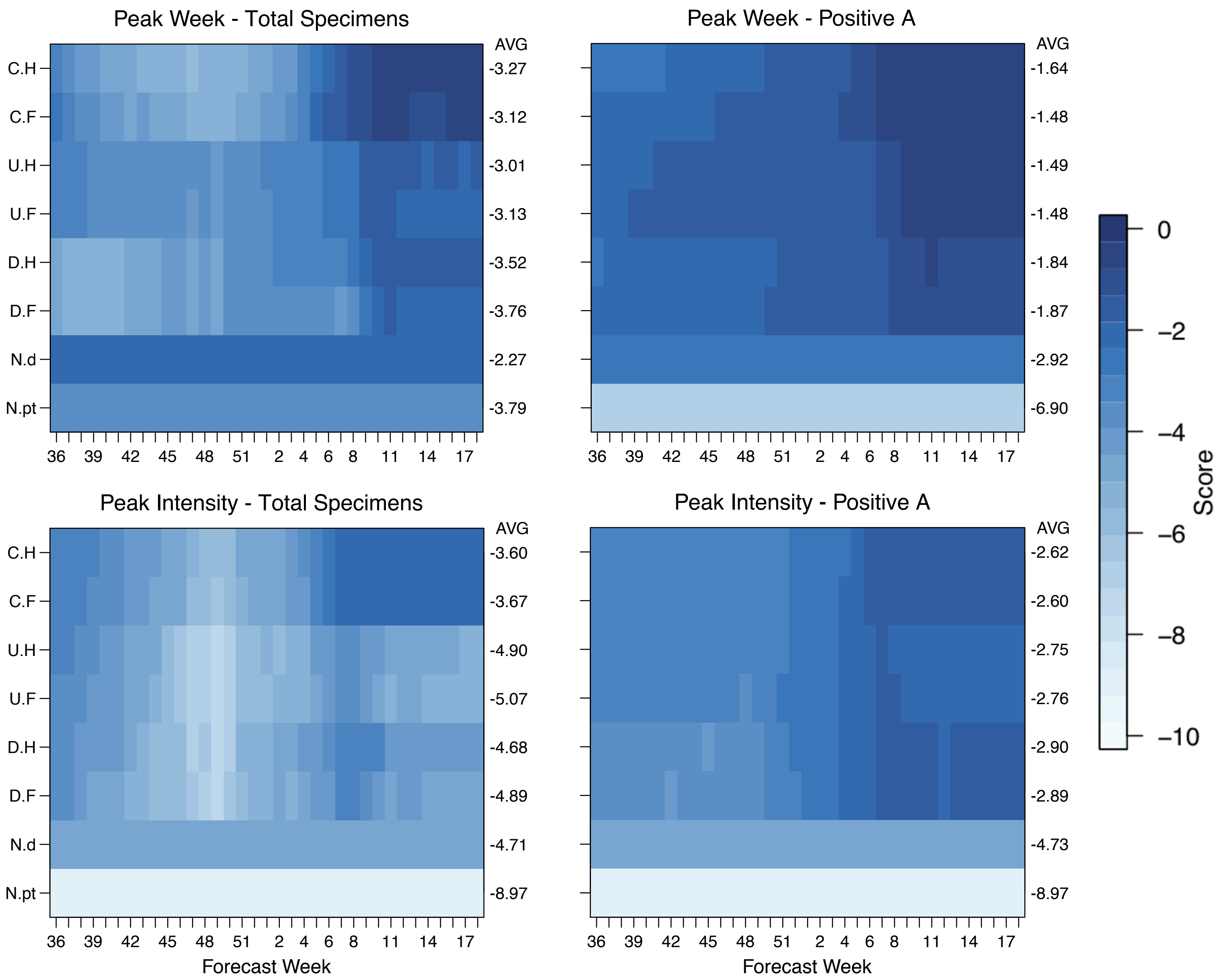

Peak Week - Total Specimens
AVG
C.H -3.27
C.F -3.12
U.H -3.01
U.F -3.13
D.H -3.52
D.F -3.76
N.d -2.27
N.pt -3.79
36 39 42 45 48 51 2 4 6 8 11 14 17
Peak Week - Positive A
AVG
-1.64
-1.48
-1.49
-1.48
-1.84
-1.87
-2.92
-6.90
36 39 42 45 48 51 2 4 6 8 11 14 17
Peak Intensity - Total Specimens
AVG
C.H -3.60
C.F -3.67
U.H -4.90
U.F -5.07
D.H -4.68
D.F -4.89
N.d -4.71
N.pt -8.97
36 39 42 45 48 51 2 4 6 8 11 14 17
Forecast Week
Peak Intensity - Positive A
AVG
-2.62
-2.60
-2.75
-2.76
-2.90
-2.89
-4.73
-8.97
36 39 42 45 48 51 2 4 6 8 11 14 17
Forecast Week
Score
0
−2
−4
−6
−8
−10
