# Supplementary material for: Accurate influenza forecasts using type-specific incidence data for small geographic units
Source: PLoS Comput Biol. 2021 Jul 29;17(7):e1009230. doi: 10.1371/journal.pcbi.1009230 (PMC8354478; doi:10.1371/journal.pcbi.1009230)
Supplement: S6 Fig — Pixel colour shows forecast single-bin score for a given observation week averaged across clusters and seasons, e.g. the 4-week ahead forecast for week 48 was made using data only up to week 44. Averages across all weeks for a given model are printed on the RHS of each row of pixels. Model type is shown on LHS y-axis. (PDF) [file pcbi.1009230.s006.pdf]

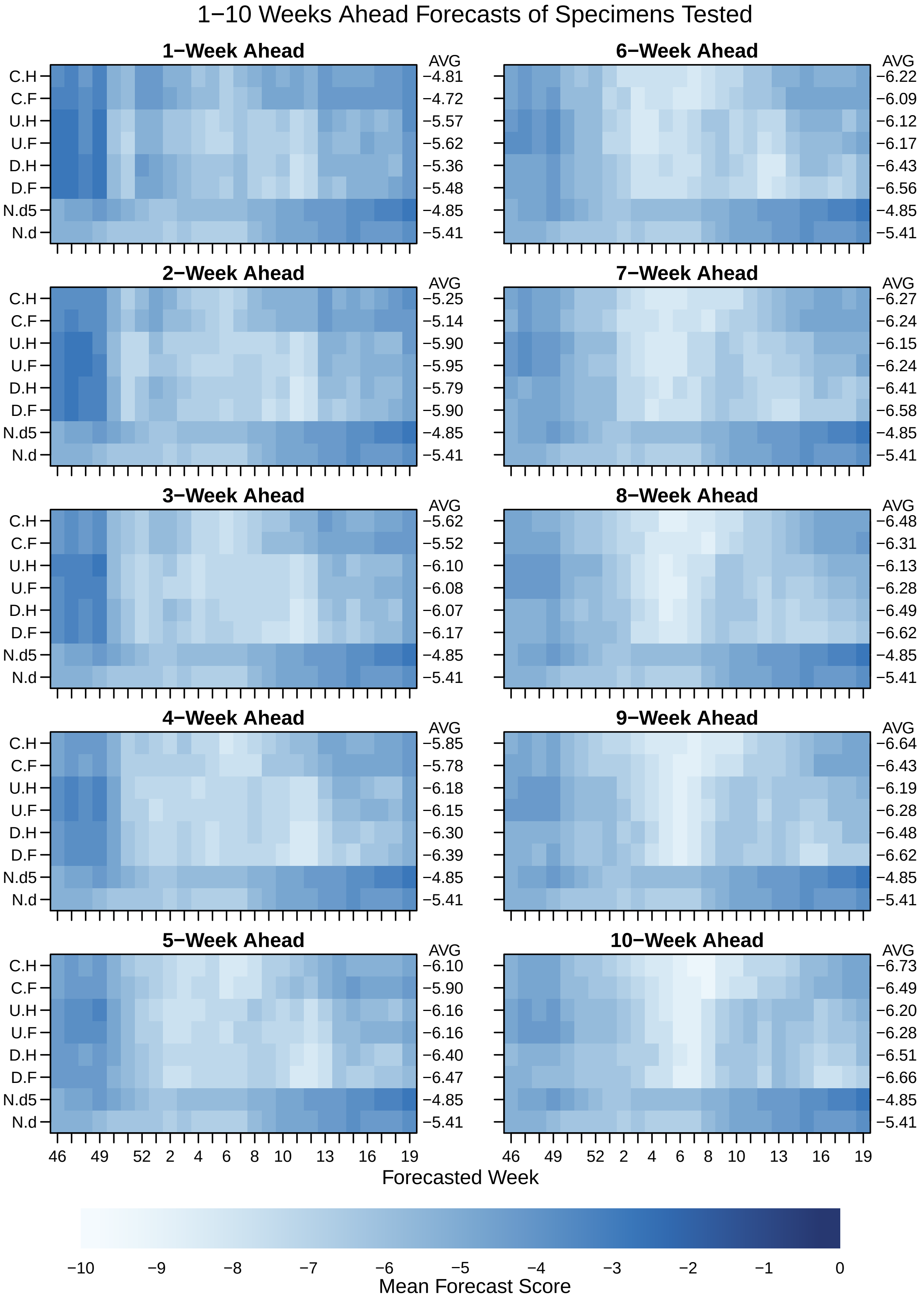

1−10 Weeks Ahead Forecasts of Specimens Tested
1−Week Ahead
AVG
C.H −4.81
C.F −4.72
U.H −5.57
U.F −5.62
D.H −5.36
D.F −5.48
N.d5 −4.85
N.d −5.41
6−Week Ahead
AVG
−6.22
−6.09
−6.12
−6.17
−6.43
−6.56
−4.85
−5.41
2−Week Ahead
AVG
C.H −5.25
C.F −5.14
U.H −5.90
U.F −5.95
D.H −5.79
D.F −5.90
N.d5 −4.85
N.d −5.41
7−Week Ahead
AVG
−6.27
−6.24
−6.15
−6.24
−6.41
−6.58
−4.85
−5.41
3−Week Ahead
AVG
C.H −5.62
C.F −5.52
U.H −6.10
U.F −6.08
D.H −6.07
D.F −6.17
N.d5 −4.85
N.d −5.41
8−Week Ahead
AVG
−6.48
−6.31
−6.13
−6.28
−6.49
−6.62
−4.85
−5.41
4−Week Ahead
AVG
C.H −5.85
C.F −5.78
U.H −6.18
U.F −6.15
D.H −6.30
D.F −6.39
N.d5 −4.85
N.d −5.41
9−Week Ahead
AVG
−6.64
−6.43
−6.19
−6.28
−6.48
−6.62
−4.85
−5.41
5−Week Ahead
AVG
C.H −6.10
C.F −5.90
U.H −6.16
U.F −6.16
D.H −6.40
D.F −6.47
N.d5 −4.85
N.d −5.41
10−Week Ahead
AVG
−6.73
−6.49
−6.20
−6.28
−6.51
−6.66
−4.85
−5.41
46 49 52 2 4 6 8 10 13 16 19
Forecasted Week
−10 −9 −8 −7 −6 −5 −4 −3 −2 −1 0
Mean Forecast Score
