# Supplementary material for: Accurate influenza forecasts using type-specific incidence data for small geographic units
Source: PLoS Comput Biol. 2021 Jul 29;17(7):e1009230. doi: 10.1371/journal.pcbi.1009230 (PMC8354478; doi:10.1371/journal.pcbi.1009230)
Supplement: S7 Fig — Pixel colour shows forecast single-bin score for a given observation week averaged across clusters and seasons, e.g. the 4-week ahead forecast for week 48 was made using data only up to week 44. Averages across all weeks for a given model are printed on the RHS of each row of pixels. Model type is shown on LHS y-axis. (PDF) [file pcbi.1009230.s007.pdf]

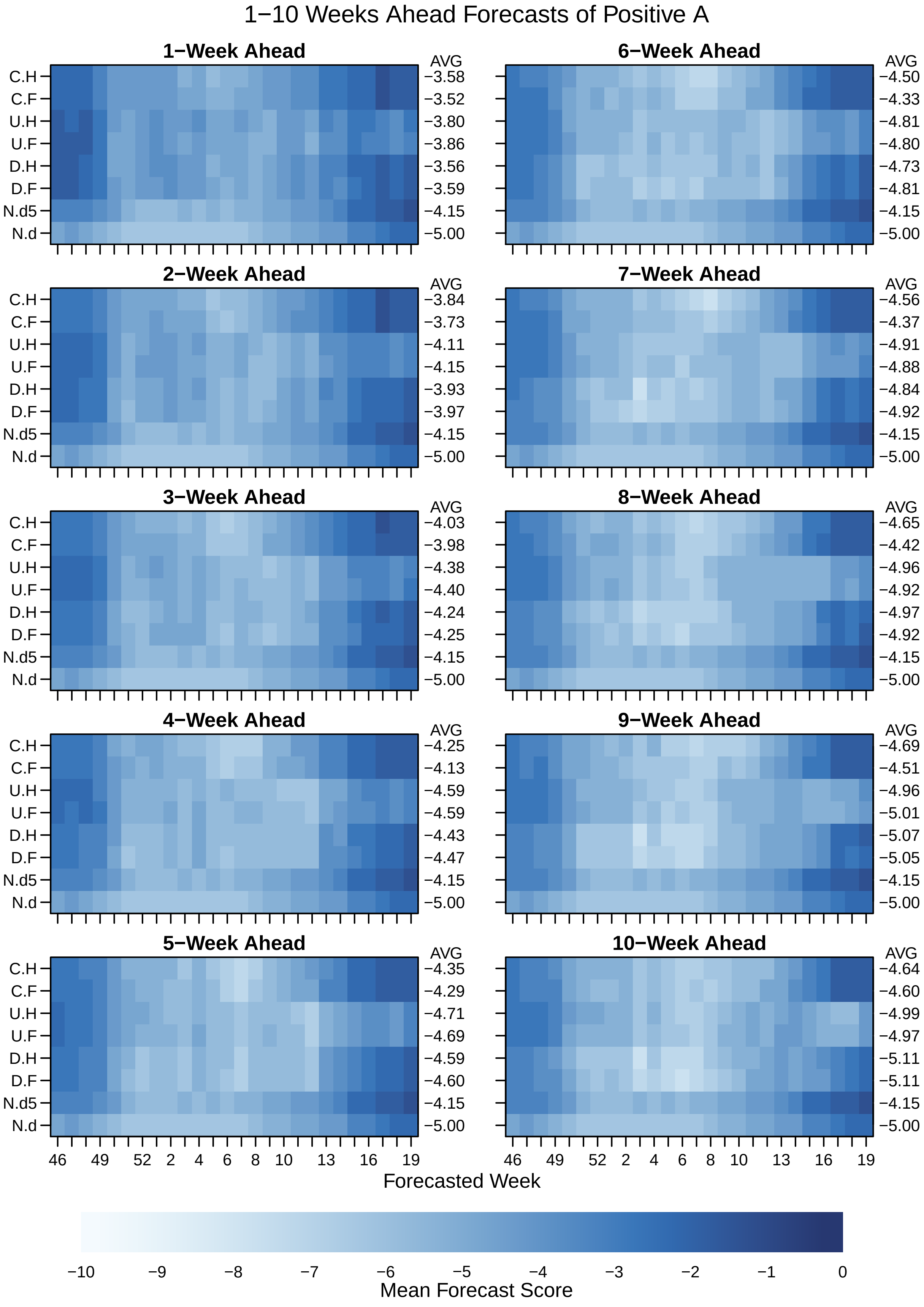
1−10 Weeks Ahead Forecasts of Positive A
1−Week Ahead
2−Week Ahead
3−Week Ahead
4−Week Ahead
5−Week Ahead
6−Week Ahead
7−Week Ahead
8−Week Ahead
9−Week Ahead
10−Week Ahead
C.H
C.F
U.H
U.F
D.H
D.F
N.d5
N.d
AVG
1-Week: −3.58 −3.52 −3.80 −3.86 −3.56 −3.59 −4.15 −5.00
2-Week: −3.84 −3.73 −4.11 −4.15 −3.93 −3.97 −4.15 −5.00
3-Week: −4.03 −3.98 −4.38 −4.40 −4.24 −4.25 −4.15 −5.00
4-Week: −4.25 −4.13 −4.59 −4.59 −4.43 −4.47 −4.15 −5.00
5-Week: −4.35 −4.29 −4.71 −4.69 −4.59 −4.60 −4.15 −5.00
6-Week: −4.50 −4.33 −4.81 −4.80 −4.73 −4.81 −4.15 −5.00
7-Week: −4.56 −4.37 −4.91 −4.88 −4.84 −4.92 −4.15 −5.00
8-Week: −4.65 −4.42 −4.96 −4.92 −4.97 −4.92 −4.15 −5.00
9-Week: −4.69 −4.51 −4.96 −5.01 −5.07 −5.05 −4.15 −5.00
10-Week: −4.64 −4.60 −4.99 −4.97 −5.11 −5.11 −4.15 −5.00
46 49 52 2 4 6 8 10 13 16 19
Forecasted Week
−10 −9 −8 −7 −6 −5 −4 −3 −2 −1 0
Mean Forecast Score
