# Supplementary material for: Accurate influenza forecasts using type-specific incidence data for small geographic units
Source: PLoS Comput Biol. 2021 Jul 29;17(7):e1009230. doi: 10.1371/journal.pcbi.1009230 (PMC8354478; doi:10.1371/journal.pcbi.1009230)
Supplement: S10 Fig — Spatial kernel κ posterior as a function of distance. Posterior distributions are across all season-years and β-modulation variants (fixed, humidity) for each cluster. Posteriors are taken from late-season fits using data through EW 18. Median kernel values appear as a black line and the 25% to 75% interval is shaded blue. For each unique county pair, there appears a vertical orange line indicating the great-circle distance (km) between population centroids. (PDF) [file pcbi.1009230.s010.pdf]

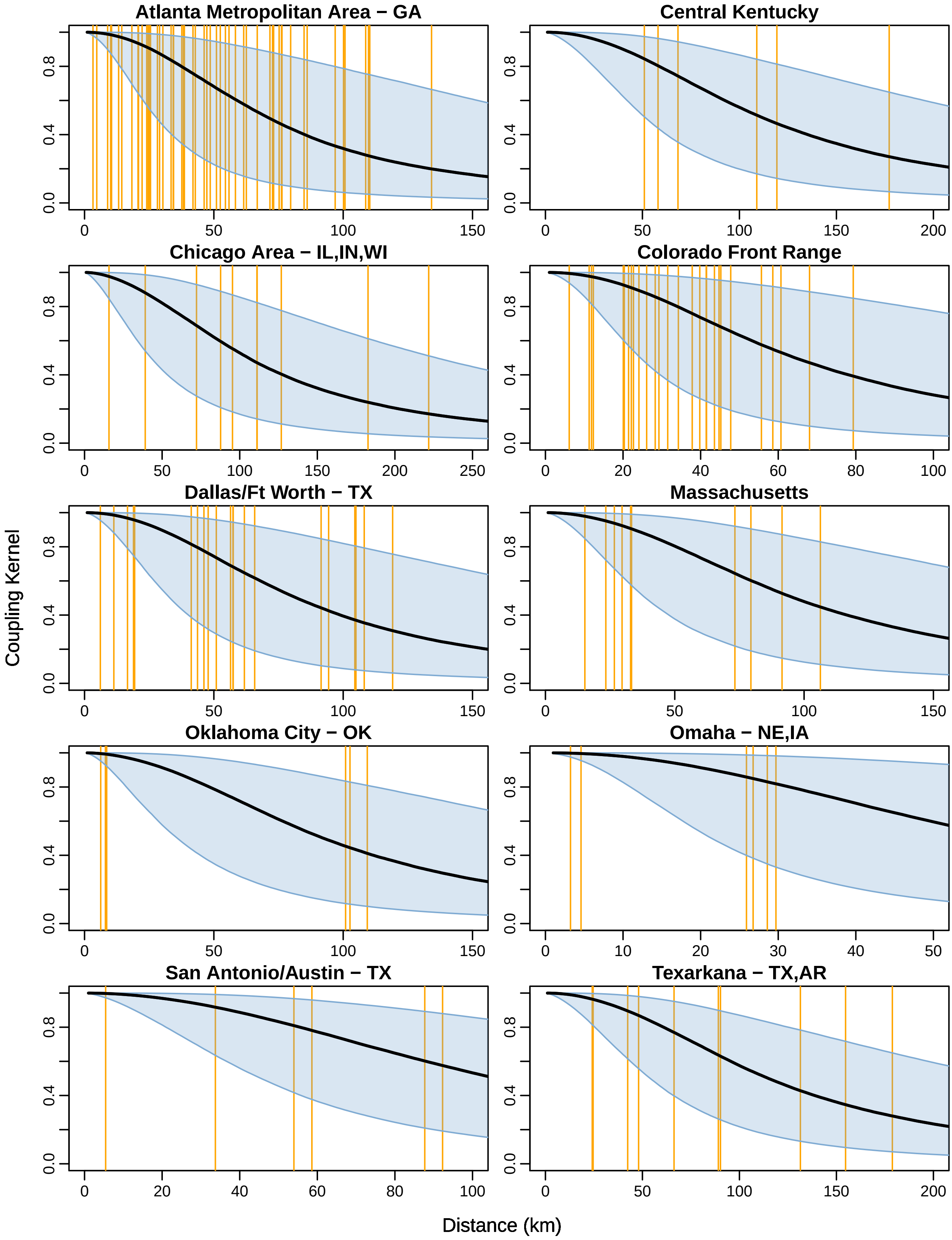

Atlanta Metropolitan Area − GA
Central Kentucky
Chicago Area − IL,IN,WI
Colorado Front Range
Dallas/Ft Worth − TX
Massachusetts
Oklahoma City − OK
Omaha − NE,IA
San Antonio/Austin − TX
Texarkana − TX,AR
Coupling Kernel
Distance (km)
