# Supplementary material for: Accurate influenza forecasts using type-specific incidence data for small geographic units
Source: PLoS Comput Biol. 2021 Jul 29;17(7):e1009230. doi: 10.1371/journal.pcbi.1009230 (PMC8354478; doi:10.1371/journal.pcbi.1009230)
Supplement: S12 Fig — Each page contains results from a single model. Rows are divided first by data metric and then by season. Columns are divided by cluster. In the panels incidence data is shown in black and shaded cones depict the central 50% forecast windows for 1–4 week ahead. Forecasts were generated for every week, but only every third forecast is shown here for clarity. Colour progression from left to right indicates time of forecast. Solid lines show median fit to data at the point the forecast was assumed to have been made. Dashed lines show forecasts beyond the time at which data were assumed to be available. Mean score is the average score across MMWR forecasted weeks and 1 to 4 weeks ahead targets. (PDF) [file pcbi.1009230.s012.pdf]

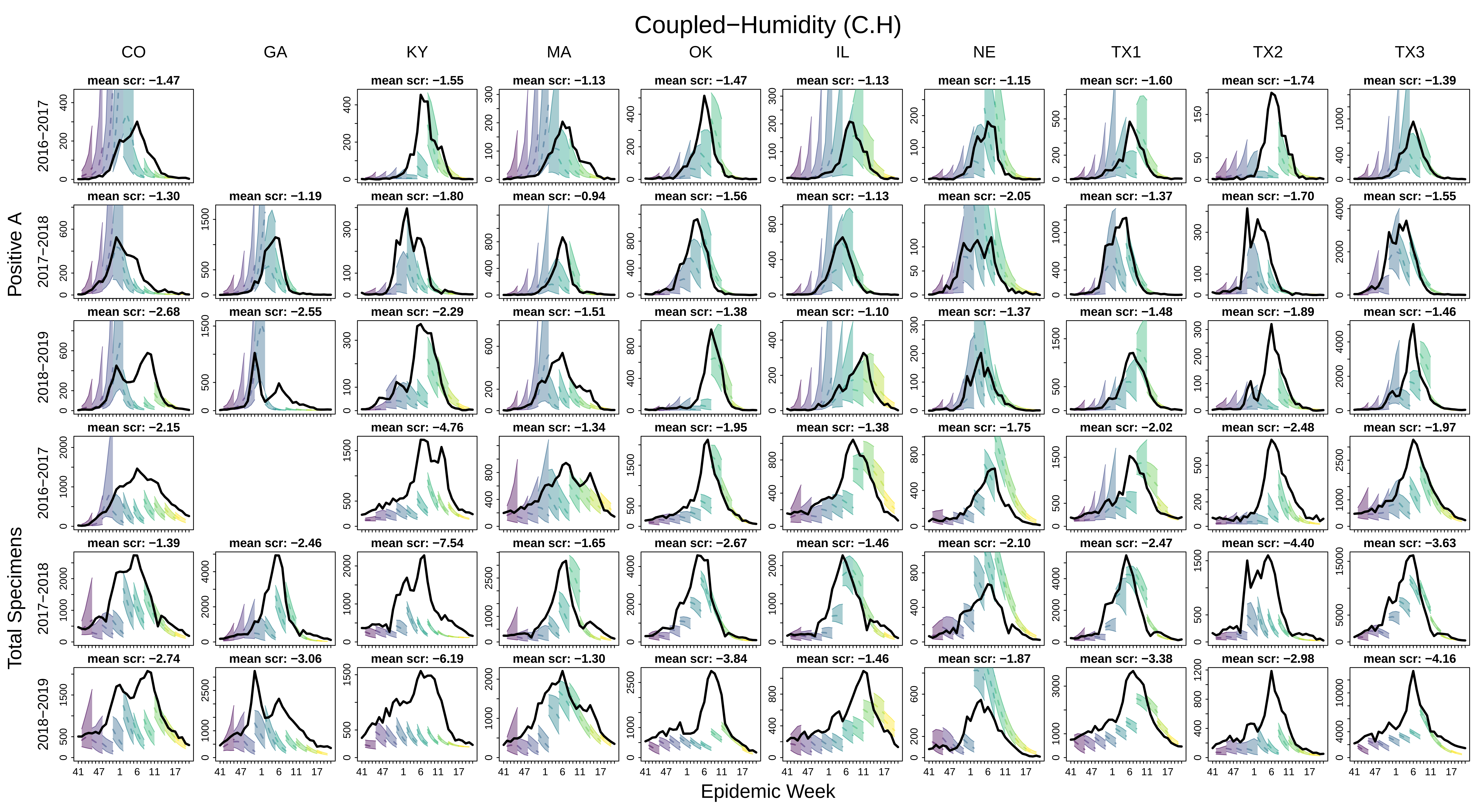
Coupled−Humidity (C.H)
CO
GA
KY
MA
OK
IL
NE
TX1
TX2
TX3
Positive A
Total Specimens
2016−2017
2017−2018
2018−2019
2016−2017
2017−2018
2018−2019
mean scr: −1.47
mean scr: −1.55
mean scr: −1.13
mean scr: −1.47
mean scr: −1.13
mean scr: −1.15
mean scr: −1.60
mean scr: −1.74
mean scr: −1.39
mean scr: −1.30
mean scr: −1.19
mean scr: −1.80
mean scr: −0.94
mean scr: −1.56
mean scr: −1.13
mean scr: −2.05
mean scr: −1.37
mean scr: −1.70
mean scr: −1.55
mean scr: −2.68
mean scr: −2.55
mean scr: −2.29
mean scr: −1.51
mean scr: −1.38
mean scr: −1.10
mean scr: −1.37
mean scr: −1.48
mean scr: −1.89
mean scr: −1.46
mean scr: −2.15
mean scr: −4.76
mean scr: −1.34
mean scr: −1.95
mean scr: −1.38
mean scr: −1.75
mean scr: −2.02
mean scr: −2.48
mean scr: −1.97
mean scr: −1.39
mean scr: −2.46
mean scr: −7.54
mean scr: −1.65
mean scr: −2.67
mean scr: −1.46
mean scr: −2.10
mean scr: −2.47
mean scr: −4.40
mean scr: −3.63
mean scr: −2.74
mean scr: −3.06
mean scr: −6.19
mean scr: −1.30
mean scr: −3.84
mean scr: −1.46
mean scr: −1.87
mean scr: −3.38
mean scr: −2.98
mean scr: −4.16
41
47
1
6
11
17
Epidemic Week

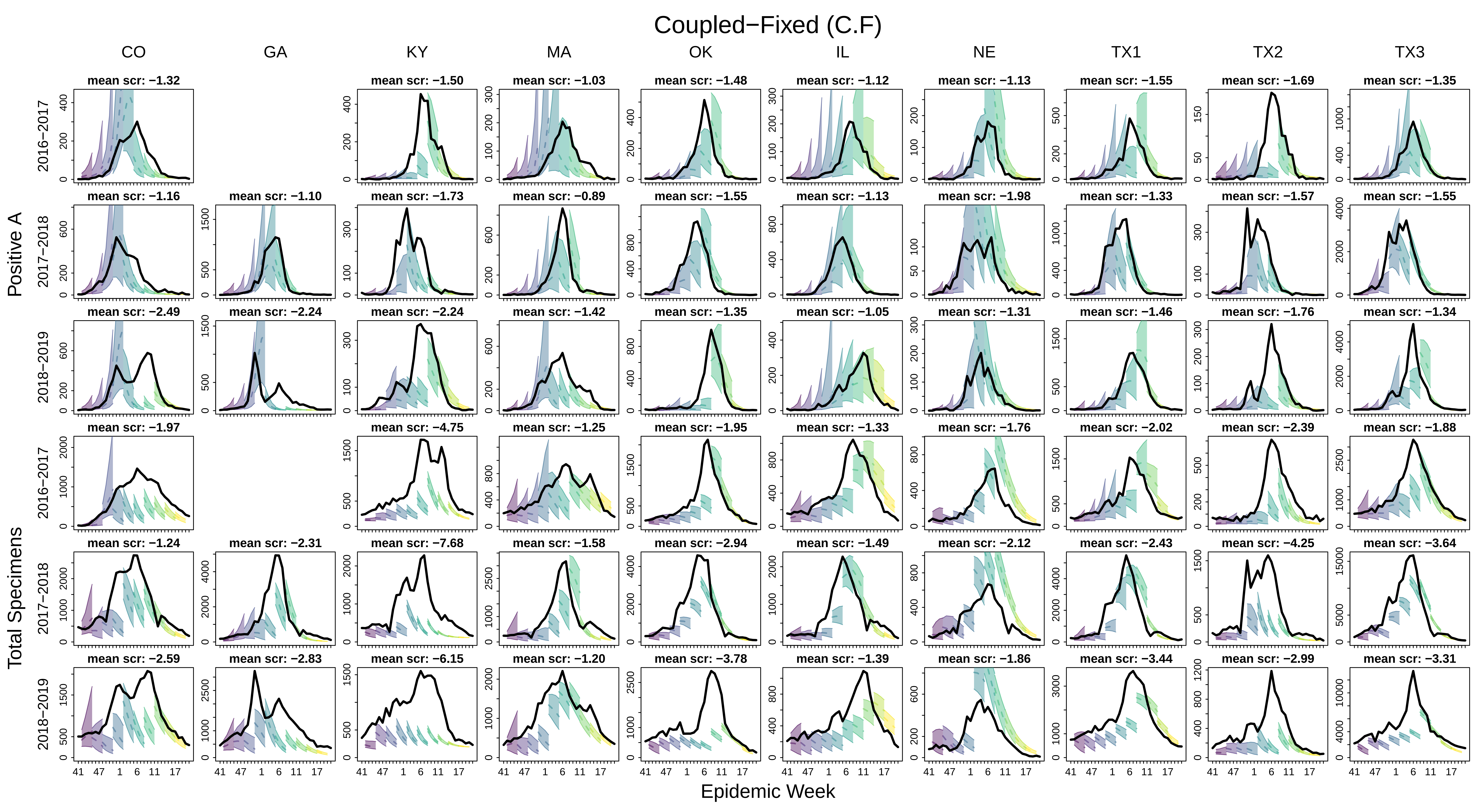

Coupled−Fixed (C.F)
CO
GA
KY
MA
OK
IL
NE
TX1
TX2
TX3
Positive A
Total Specimens
2016−2017
2017−2018
2018−2019
mean scr: −1.32
mean scr: −1.50
mean scr: −1.03
mean scr: −1.48
mean scr: −1.12
mean scr: −1.13
mean scr: −1.55
mean scr: −1.69
mean scr: −1.35
mean scr: −1.16
mean scr: −1.10
mean scr: −1.73
mean scr: −0.89
mean scr: −1.55
mean scr: −1.13
mean scr: −1.98
mean scr: −1.33
mean scr: −1.57
mean scr: −1.55
mean scr: −2.49
mean scr: −2.24
mean scr: −2.24
mean scr: −1.42
mean scr: −1.35
mean scr: −1.05
mean scr: −1.31
mean scr: −1.46
mean scr: −1.76
mean scr: −1.34
mean scr: −1.97
mean scr: −4.75
mean scr: −1.25
mean scr: −1.95
mean scr: −1.33
mean scr: −1.76
mean scr: −2.02
mean scr: −2.39
mean scr: −1.88
mean scr: −1.24
mean scr: −2.31
mean scr: −7.68
mean scr: −1.58
mean scr: −2.94
mean scr: −1.49
mean scr: −2.12
mean scr: −2.43
mean scr: −4.25
mean scr: −3.64
mean scr: −2.59
mean scr: −2.83
mean scr: −6.15
mean scr: −1.20
mean scr: −3.78
mean scr: −1.39
mean scr: −1.86
mean scr: −3.44
mean scr: −2.99
mean scr: −3.31
41 47 1 6 11 17
Epidemic Week

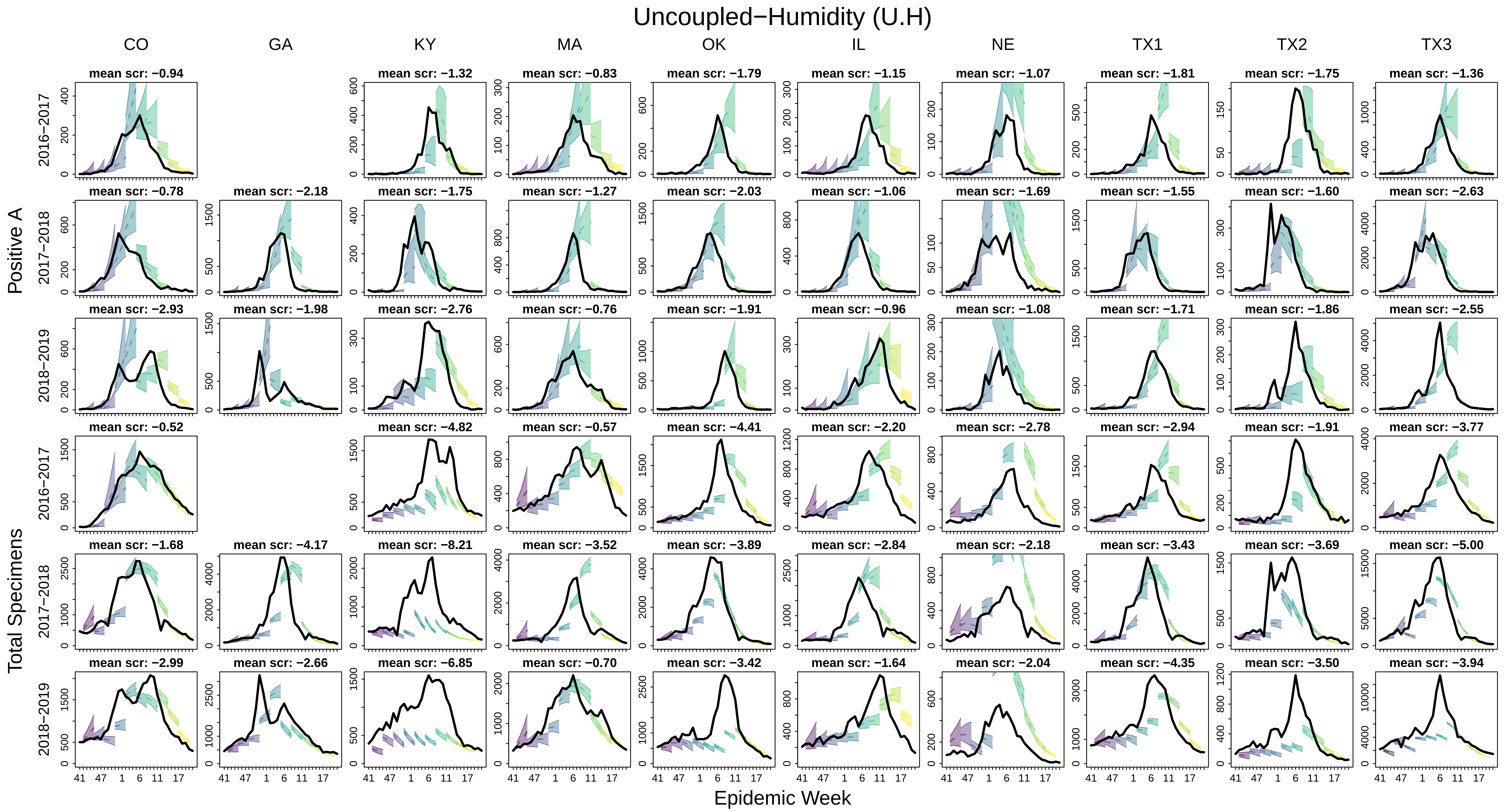
Uncoupled−Humidity (U.H)
CO
GA
KY
MA
OK
IL
NE
TX1
TX2
TX3
Positive A
Total Specimens
2016−2017
2017−2018
2018−2019
2016−2017
2017−2018
2018−2019
mean scr: −0.94
mean scr: −1.32
mean scr: −0.83
mean scr: −1.79
mean scr: −1.15
mean scr: −1.07
mean scr: −1.81
mean scr: −1.75
mean scr: −1.36
mean scr: −0.78
mean scr: −2.18
mean scr: −1.75
mean scr: −1.27
mean scr: −2.03
mean scr: −1.06
mean scr: −1.69
mean scr: −1.55
mean scr: −1.60
mean scr: −2.63
mean scr: −2.93
mean scr: −1.98
mean scr: −2.76
mean scr: −0.76
mean scr: −1.91
mean scr: −0.96
mean scr: −1.08
mean scr: −1.71
mean scr: −1.86
mean scr: −2.55
mean scr: −0.52
mean scr: −4.82
mean scr: −0.57
mean scr: −4.41
mean scr: −2.20
mean scr: −2.78
mean scr: −2.94
mean scr: −1.91
mean scr: −3.77
mean scr: −1.68
mean scr: −4.17
mean scr: −8.21
mean scr: −3.52
mean scr: −3.89
mean scr: −2.84
mean scr: −2.18
mean scr: −3.43
mean scr: −3.69
mean scr: −5.00
mean scr: −2.99
mean scr: −2.66
mean scr: −6.85
mean scr: −0.70
mean scr: −3.42
mean scr: −1.64
mean scr: −2.04
mean scr: −4.35
mean scr: −3.50
mean scr: −3.94
41 47 1 6 11 17
Epidemic Week

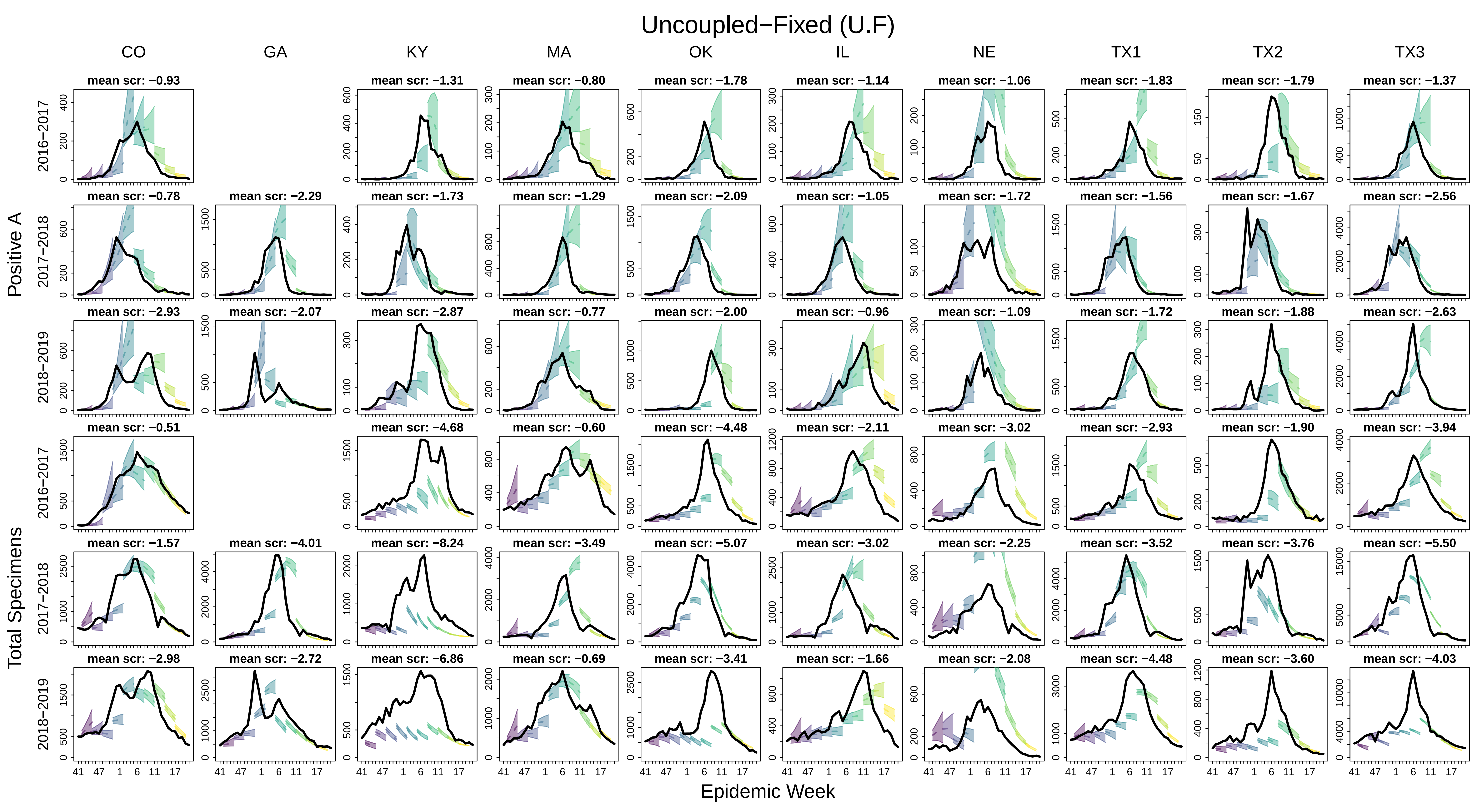

Uncoupled−Fixed (U.F)
CO
GA
KY
MA
OK
IL
NE
TX1
TX2
TX3
Positive A
Total Specimens
2016−2017
2017−2018
2018−2019
mean scr: −0.93
mean scr: −1.31
mean scr: −0.80
mean scr: −1.78
mean scr: −1.14
mean scr: −1.06
mean scr: −1.83
mean scr: −1.79
mean scr: −1.37
mean scr: −0.78
mean scr: −2.29
mean scr: −1.73
mean scr: −1.29
mean scr: −2.09
mean scr: −1.05
mean scr: −1.72
mean scr: −1.56
mean scr: −1.67
mean scr: −2.56
mean scr: −2.93
mean scr: −2.07
mean scr: −2.87
mean scr: −0.77
mean scr: −2.00
mean scr: −0.96
mean scr: −1.09
mean scr: −1.72
mean scr: −1.88
mean scr: −2.63
mean scr: −0.51
mean scr: −4.68
mean scr: −0.60
mean scr: −4.48
mean scr: −2.11
mean scr: −3.02
mean scr: −2.93
mean scr: −1.90
mean scr: −3.94
mean scr: −1.57
mean scr: −4.01
mean scr: −8.24
mean scr: −3.49
mean scr: −5.07
mean scr: −3.02
mean scr: −2.25
mean scr: −3.52
mean scr: −3.76
mean scr: −5.50
mean scr: −2.98
mean scr: −2.72
mean scr: −6.86
mean scr: −0.69
mean scr: −3.41
mean scr: −1.66
mean scr: −2.08
mean scr: −4.48
mean scr: −3.60
mean scr: −4.03
41 47 1 6 11 17
Epidemic Week

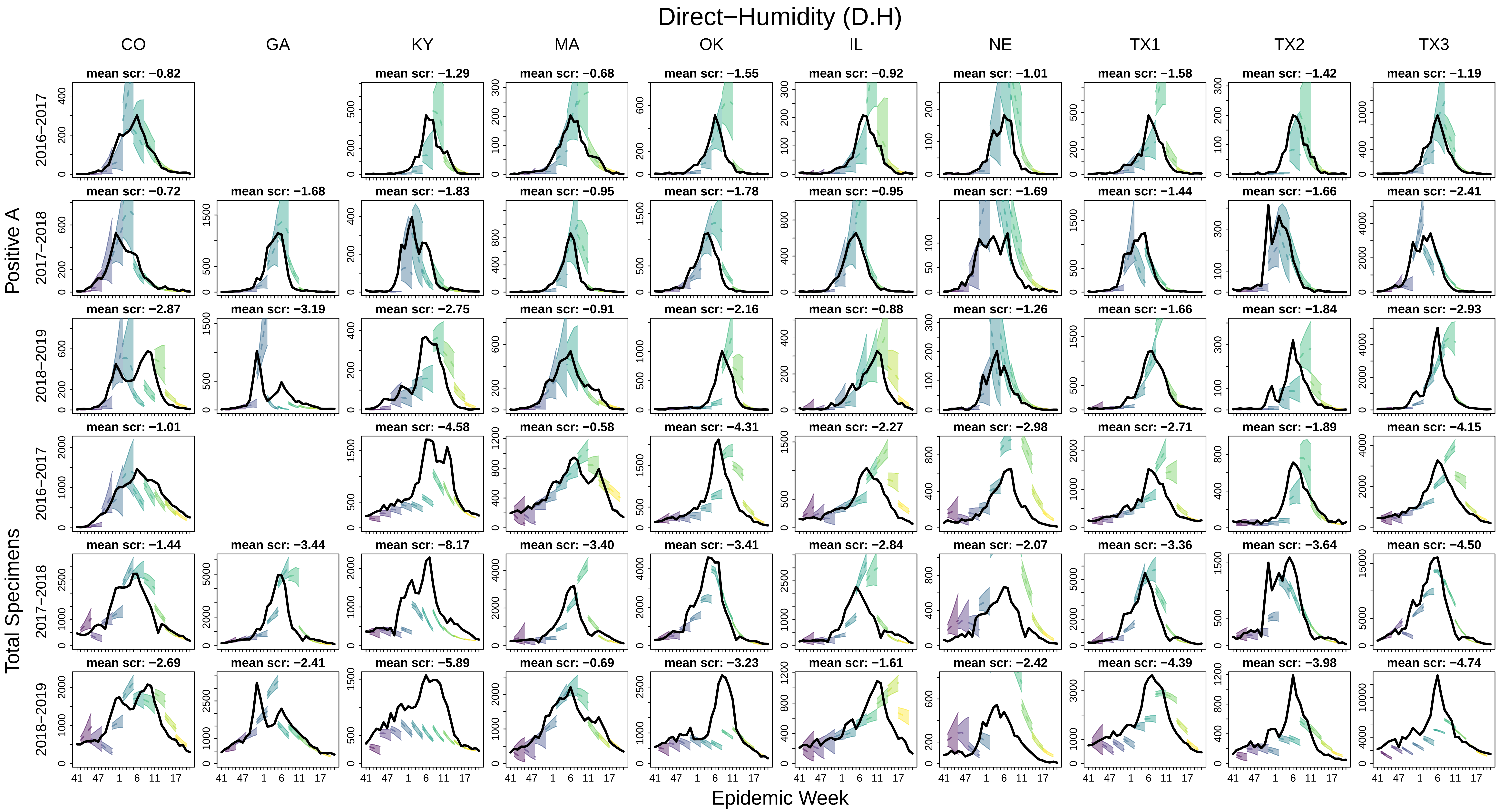
Direct−Humidity (D.H)
CO
GA
KY
MA
OK
IL
NE
TX1
TX2
TX3
Positive A
Total Specimens
2016−2017
2017−2018
2018−2019
mean scr: −0.82
mean scr: −1.29
mean scr: −0.68
mean scr: −1.55
mean scr: −0.92
mean scr: −1.01
mean scr: −1.58
mean scr: −1.42
mean scr: −1.19
mean scr: −0.72
mean scr: −1.68
mean scr: −1.83
mean scr: −0.95
mean scr: −1.78
mean scr: −0.95
mean scr: −1.69
mean scr: −1.44
mean scr: −1.66
mean scr: −2.41
mean scr: −2.87
mean scr: −3.19
mean scr: −2.75
mean scr: −0.91
mean scr: −2.16
mean scr: −0.88
mean scr: −1.26
mean scr: −1.66
mean scr: −1.84
mean scr: −2.93
mean scr: −1.01
mean scr: −4.58
mean scr: −0.58
mean scr: −4.31
mean scr: −2.27
mean scr: −2.98
mean scr: −2.71
mean scr: −1.89
mean scr: −4.15
mean scr: −1.44
mean scr: −3.44
mean scr: −8.17
mean scr: −3.40
mean scr: −3.41
mean scr: −2.84
mean scr: −2.07
mean scr: −3.36
mean scr: −3.64
mean scr: −4.50
mean scr: −2.69
mean scr: −2.41
mean scr: −5.89
mean scr: −0.69
mean scr: −3.23
mean scr: −1.61
mean scr: −2.42
mean scr: −4.39
mean scr: −3.98
mean scr: −4.74
41 47 1 6 11 17
Epidemic Week

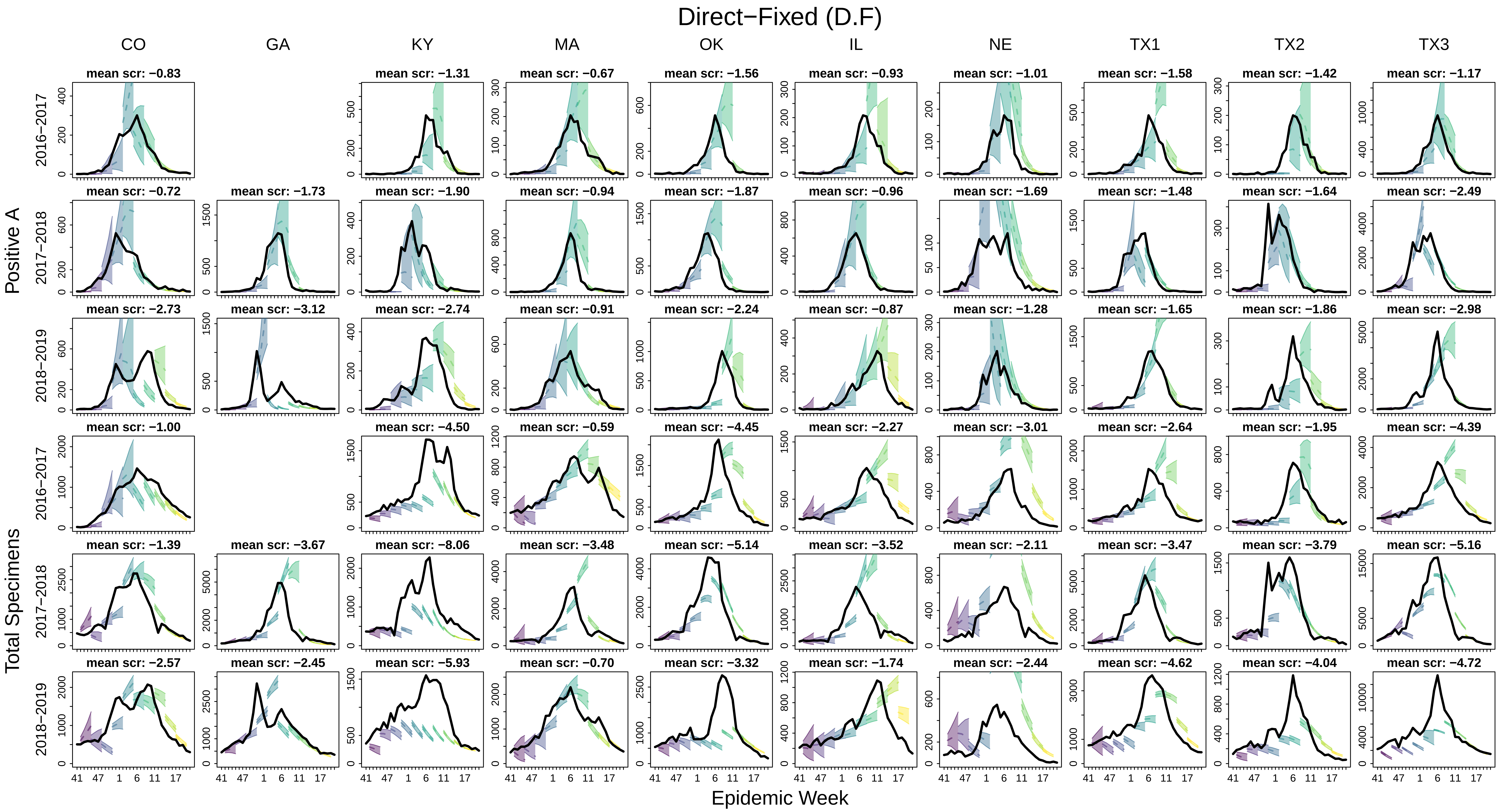

Direct−Fixed (D.F)
CO
GA
KY
MA
OK
IL
NE
TX1
TX2
TX3
Positive A
Total Specimens
2016−2017
2017−2018
2018−2019
mean scr: −0.83
mean scr: −1.31
mean scr: −0.67
mean scr: −1.56
mean scr: −0.93
mean scr: −1.01
mean scr: −1.58
mean scr: −1.42
mean scr: −1.17
mean scr: −0.72
mean scr: −1.73
mean scr: −1.90
mean scr: −0.94
mean scr: −1.87
mean scr: −0.96
mean scr: −1.69
mean scr: −1.48
mean scr: −1.64
mean scr: −2.49
mean scr: −2.73
mean scr: −3.12
mean scr: −2.74
mean scr: −0.91
mean scr: −2.24
mean scr: −0.87
mean scr: −1.28
mean scr: −1.65
mean scr: −1.86
mean scr: −2.98
mean scr: −1.00
mean scr: −4.50
mean scr: −0.59
mean scr: −4.45
mean scr: −2.27
mean scr: −3.01
mean scr: −2.64
mean scr: −1.95
mean scr: −4.39
mean scr: −1.39
mean scr: −3.67
mean scr: −8.06
mean scr: −3.48
mean scr: −5.14
mean scr: −3.52
mean scr: −2.11
mean scr: −3.47
mean scr: −3.79
mean scr: −5.16
mean scr: −2.57
mean scr: −2.45
mean scr: −5.93
mean scr: −0.70
mean scr: −3.32
mean scr: −1.74
mean scr: −2.44
mean scr: −4.62
mean scr: −4.04
mean scr: −4.72
41 47 1 6 11 17
Epidemic Week
