# Supplementary material for: Accurate influenza forecasts using type-specific incidence data for small geographic units
Source: PLoS Comput Biol. 2021 Jul 29;17(7):e1009230. doi: 10.1371/journal.pcbi.1009230 (PMC8354478; doi:10.1371/journal.pcbi.1009230)
Supplement: S1 Table — Correlations are calculated for each cluster and season, and comparing total specimens, positive for, A and CDC %ILI. From the resulting distributions, we have extracted the median, 20%, and 80% quantiles. (PDF) [file pcbi.1009230.s013.pdf]

Metrics Compared	Median	20%	80%
CDC %ILI v Total Spec	0.955	0.928	0.973
CDC %ILI v Positive A	0.924	0.860	0.945
Total Spec v Pos A	0.937	0.911	0.958
